# Transcriptome Analysis of Cardiac Hypertrophic Growth in *MYBPC3*-Null Mice Suggests Early Responders in Hypertrophic Remodeling

**DOI:** 10.3389/fphys.2018.01442

**Published:** 2018-10-25

**Authors:** Emily Farrell, Annie E. Armstrong, Adrian C. Grimes, Francisco J. Naya, Willem J. de Lange, J. Carter Ralphe

**Affiliations:** ^1^Department of Pediatrics, University of Wisconsin School of Medicine and Public Health, Madison, WI, United States; ^2^Department of Biology, Boston University, Boston, MA, United States

**Keywords:** hypertrophic cardiomyopathy, HCM, cardiac myosin-binding protein C, Xirp2, *Kcnd2*, K_v_4.2, *Plzf*, *Zbtb16*

## Abstract

**Rationale:** With a prevalence of 1 in 200 individuals, hypertrophic cardiomyopathy (HCM) is thought to be the most common genetic cardiac disease, with potential outcomes that include severe hypertrophy, heart failure, and sudden cardiac death (SCD). Though much research has furthered our understanding of how HCM-causing mutations in genes such as cardiac myosin-binding protein C (*MYBPC3*) impair contractile function, it remains unclear how such dysfunction leads to hypertrophy and/or arrhythmias, which comprise the HCM phenotype. Identification of early response mediators could provide rational therapeutic targets to reduce disease severity. Our goal was to differentiate physiologic and pathophysiologic hypertrophic growth responses and identify early genetic mediators in the development of cardiomegaly in the cardiac myosin-binding protein C-null (cMyBP-C^-/-^) mouse model of HCM.

**Methods and Results:** We performed microarray analysis on left ventricles of wild-type (WT) and cMyBPC^-/-^ mice (*n* = 7 each) at postnatal day (PND) 1 and PND 9, before and after the appearance of an overt HCM phenotype. Applying the criteria of ≥2-fold change, we identified genes whose change was exclusive to pathophysiologic growth (*n* = 61), physiologic growth (*n* = 30), and genes whose expression changed ≥2-fold in both WT and cMyBP-C^-/-^ hearts (*n* = 130). Furthermore, we identified genes that were dysregulated in PND1 cMyBP-C^-/-^ hearts prior to hypertrophy, including genes in mechanosensing pathways and potassium channels linked to arrhythmias. One gene of interest, *Xirp2*, and its protein product, are regulated during growth but also show early, robust prehypertrophic upregulation in cMyBP-C^-/-^ hearts. Additionally, the transcription factor Zbtb16 also shows prehypertrophic upregulation at both gene and protein levels.

**Conclusion:** Our transcriptome analysis generated a comprehensive data set comparing physiologic vs. hypertrophic growth in mice lacking cMyBP-C. It highlights the importance of extracellular matrix pathways in hypertrophic growth and early dysregulation of potassium channels. Prehypertrophic upregulation of Xirp2 in cMyBP-C^-/-^ hearts supports a growing body of evidence suggesting Xirp2 has the capacity to elicit both hypertrophy and arrhythmias in HCM. Dysregulation of *Xirp2*, as well as *Zbtb16*, along with other genes associated with mechanosensing regions of the cardiomyocyte implicate stress-sensing in these regions as a potentially important early response in HCM.

## Introduction

Hypertrophic cardiomyopathy (HCM), now thought to be the leading genetic cardiovascular disease (Semsarian et al., 2015), is characterized by hypertrophy of the left ventricular free wall and septum and can be accompanied by myocardial disarray, diastolic dysfunction, and fibrosis (Fananapazir and Epstein, 1994; Davies and McKenna, 1995). HCM is also the most common cause of sudden cardiac death (SCD) (Maron et al., 2006), which results from lethal arrhythmias. Genes encoding the sarcomeric proteins β-myosin heavy chain (β-MHC) and cardiac myosin-binding protein C (cMyBP-C) harbor the majority of HCM-causing mutations in this autosomal dominant disease (Richard et al., 2003; Olivotto et al., 2008; Alfares et al., 2015). The cellular signaling processes that lead from the primary sarcomeric mutation to the hypertrophic and/or arrhythmic phenotype remain poorly understood, making disease prognosis difficult to predict and targeted treatments elusive. Furthermore, hypertrophy and arrhythmias can occur independently in HCM, further complicating the ability to uncover the cellular signaling pathways (Moolman et al., 1997; Christiaans et al., 2009).

Cardiac myosin-binding protein C is an adrenergic-responsive regulator of cardiac contractility, largely responsible for modulating cardiac output to meet increased demand. The majority of mutations in the *MYBPC3* gene lead to decreased levels of functional cMyBP-C and, in animal models, result in accelerated sarcomere cross-bridge cycling and increased contractile kinetics. A mouse in which cMyBP-C is genetically ablated (cMyBP-C^-/-^) has been widely used as a model of HCM (Harris et al., 2002; Merkulov et al., 2012; de Lange et al., 2013; Farrell et al., 2017; Toib et al., 2017). At birth, cMyBP-C^-/-^ hearts are virtually indistinguishable from hearts of wild-type (WT) littermates, but by postnatal day (PND) 9, they are overtly hypertrophic and exhibit systolic and diastolic dysfunction (de Lange et al., 2013; Farrell et al., 2017). To probe the etiology of the hypertrophic signaling, we performed microarray analysis on cMyBP-C^-/-^ left ventricles both prior (PND1) and after (PND9) the development of hypertrophy. Our initial analysis of the microarray data focused on global pathway alterations in the perinatal, pre-hypertrophic cMyBP-C^-/-^ heart and revealed increased cardiomyocyte cell cycling and proliferation (Farrell et al., 2017). Indeed, increased cell cycling (Jiang et al., 2015; Nixon et al., 2017) and cardiomyocyte proliferation (Jiang et al., 2015) in the early postnatal period has been observed in other models in which cMyBP-C is ablated. Here, we focus our analysis on identification of individual genes whose dysregulation might substantially contribute to early disease progression. In addition, we distinguished gene expression programs involved in physiologic vs. pathophysiologic/hypertrophic growth.

The results of this study identify differences in gene expression between physiologic cardiac growth and pathologic cardiac growth. The microarray highlights extracellular matrix and structural pathways as uniquely modulated in hypertrophic growth, and several potassium channels with pre- and post-hypertrophic dysregulation. We also identified upregulation of *Xirp2* (aka *Cmya5, mXinβ*, and *Myomaxin*) both prior to the development of the HCM phenotype and also at PND9 in the cMyBP-C^-/-^ vs. WT left ventricles. Xirp2, encoded by the *Xirp2* gene, has been shown to localize to mechanosensing regions of the cardiomyocyte including the intercalated disc (Wang et al., 2010), z-disc (Huang et al., 2006; Eulitz et al., 2013), and costameres (Huang et al., 2006). Dysregulation of *Xirp2* is linked to both cardiomyopathy (Duka et al., 2006; McCalmon et al., 2010; Wang et al., 2014; Long et al., 2015) and arrhythmogenesis (Huang et al., 2018). In the current study the upregulation of *Xirp2* and a transcription factor, *Zbtb16*, in the cMyBP-C^-/-^ mouse was accompanied by the dysregulation of other genes whose proteins also localize to mechanosensing regions of the cardiomyocyte or are involved in mechanosensing pathways. Together, these data lend additional support for the role of mechanosensing as an early mediator along the pathway to hypertrophy in HCM (Cox et al., 2008; Lyon et al., 2015).

## Materials and Methods

### Animals

Heterozygous (cMyBP-C^+/-^) adults were derived from backcrosses of WT E129X1/SvJ mice (Taconic, Hudson, NY, United States) with homozygous cMyBP-C^-/-^ animals previously generated on the E129X1/SvJ background (Harris et al., 2002). Subsequent breedings of cMyBP-C^+/-^ adults resulted in expected Mendelian frequencies (1:2:1) of WT (cMyBP-C^+/+^), heterozygous (cMyBP-C^+/-^), and homozygous knockout (cMyBP-C^-/-^) animals. Mice used for immunohistochemistry and neonatal qPCR were derived from cMyBP-C^+/-^ crossings, with subsequent postmortem genotyping of pups. Littermate comparisons among genotypes were performed whenever possible. No statistical differences in qPCR and western blotting results were found between hearts from cMyBP-C heterozygote (cMyBP-C^+/-^) crossings and those from WT × WT or cMyBP-C^-/-^ × cMyBP-C^-/-^ breedings; thus, the latter were used for adult qPCR and western blotting experiments. To generate embryonic day (E)18.5 pups, cMyBP-C^+/-^ dams were bred to cMyBP-C^+/-^ males for 24 h, after which mice were separated to establish timing of pregnancy. E18.5 pup hearts were harvested on day 19 after the breeding date.

Animals were anaesthetized using inhaled isoflurane (adult mice) or decapitated (embryonic and neonatal pups) and hearts were harvested. All hearts were rinsed in 1× PBS, and the blood ejected using blunt forceps. Hearts were then weighed and snap frozen in liquid nitrogen. A post-mortem tail snip was taken from each mouse of cMyBP-C^+/-^ × cMyBP-C^+/-^ breedings, to allow subsequent genotyping and sex determination by PCR. This study was approved by the Animal Care and Use Committee of the School of Medicine and Public Health at the University of Wisconsin Madison in accordance with the Guide for the Care and Use of Laboratory Animals (National Institutes of Health publication no. 85-23, revised 1985).

### Microarray Hybridization and Data Analysis

The left ventricular free wall was extracted from hearts of WT and cMyBP-C^-/-^ pups at PND1 and PND9 (*n* = 7 males for each genotype/age) and snap frozen in liquid nitrogen. After determination of genotype and sex, RNA was isolated using Trizol reagent (Invitrogen) and the Qiagen RNeasy kit. Quantity, quality, and integrity of RNA was determined using NanoDrop 2000 (Thermo Scientific), Agilent BioAnalyzer, and Agilent RNA Nano Chip (Agilent Technologies). 400 ng RNA (and 400 ng of poly-A RNA control) was used for overnight labeling with GeneChip WT Expression kit (Ambion) according to the manufacturer’s protocols. Following labeling, samples were quantified on NanoDrop 2000 and 10 μg of purified cRNA was used to generate single strand cDNA, 5.5 μg of which was then fragmented and subjected to end-terminus labeling using WT Terminal Labeling kit (Affymetrix). Samples were hybridized to Affymetrix Mouse Gene 1.0 ST whole genome arrays at 45°C for 16 h following manufacturer’s protocol. Arrays were post-processed on the Affymetrix GeneChip Fluidics Station 450, scanned using GeneChip 3000 7G, and data extracted and processed using Affymetrix Command Console (version 3.1.1.1229). Resulting GeneChip.cel files were uploaded to iReport (Ingenuity Systems) or Genesifter (Geospiza), and normalized *via* robust multi-array average (RMA) using default options of each system. See Section “Statistical Analysis” for details of statistical analysis.

### RT-qPCR

Whole hearts were dissected from WT and cMyBP-C^-/-^ pups of ages E18.5, PND0, PND1, PND2, PND9, and adult (13-19 weeks), weighed after removal of great vessels, and snap frozen in liquid nitrogen. RNA was isolated as described previously (Farrell et al., 2017) and detailed in Supplementary Methods. Hearts allocated for RT-qPCR remained with atria intact to avoid an additional dissection after weighing, which would increase the risk for RNA degradation. Comparison of qPCR and microarray data was performed to assess any differences that could be due to the inclusion of atria. Assays used in qPCR are listed in Supplementary Methods.

### Cryopreservation, Sectioning, and Immunohistochemistry

Hearts were harvested from WT and cMyBP-C^-/-^ pups at PND1 and cryopreserved according to standard procedures as detailed in Supplementary Methods. WT and cMyBP-C^-/-^ hearts were then sectioned in a coronal plane at 6 μm thickness (CRYO 03/5800), mounted onto charged slides (TruBond 380), fixed in 100% acetone for 15 min, and dried. Slides were rehydrated in TBS, then permeabilized with 20 ng/ml Digitonin (Sigma) in TBS and blocked in 5% normal goat serum in TBST for 2 h. Sections were incubated with primary antibodies overnight at 4°C with anti-sarcomeric α-actinin (1:1,000, Sigma, A7811) and either anti-Connexin-43 (1:200, Sigma, C6219), anti-Xirp2 (1:500, ProteinTech, 11896-1-AP), or anti-N-cadherin (1:200, ThermoScientific, CDH2 3B9). Slides were incubated with secondary antibodies (Alexa Fluor 647 and 488; Invitrogen) at 1:1,000 for 1 h at room temperature. Following labeling, sections were coverslipped using Prolong Gold Antifade Reagent (Invitrogen) with 4′,6-diamidino-2-phenylindole (DAPI) to label nuclei. Imaging was performed using Nikon Eclipse 90i photomicroscope using the S Fluor 40× objective, and NIS Elements imaging software (version 4.0; Nikon).

### Protein Harvest and Western Blot Analysis

For western blot analysis of Xirp2 protein expression, hearts were excised from WT and cMyBP-C^-/-^ mice at PND1, PND9, and adult time points, rinsed in DPBS (Thermo Fisher Scientific), atria and aorta removed, blotted dry, and weighed. Ventricles were minced and homogenized in buffer containing 20 mM HEPES (pH 7.2) (Sigma), 25 mM NaCl (Sigma), 2 mM EGTA (Sigma), 1% Triton X-100 (Sigma), 1% protease inhibitor, and 1% phosphatase inhibitor as described (Wang et al., 2010) with modifications as detailed. Buffer volume was added in proportion to heart weight such that mg-ventricle weight per volume of buffer was equal between hearts. Due to the labile nature of Xirp2, a proportional volume of 3× SDS-PAGE gel sample buffer was added to the fresh homogenate. Aliquots were then stored at -80°C until use. Additional striated and non-striated muscles, tongue, and spleen were homogenized as described above and used as positive and negative controls, respectively. A volume of homogenate equivalent to 300 μg of wet ventricle weight was fractionated in 4–15% SDS-PAGE gels (Bio-Rad) and transferred overnight at 15 V in Tris–glycine buffer with 0.02% SDS (Bio-Rad) and 20% methanol. Immunoblotting for Xirp2 was performed using unpurified BSU2 anti-myomaxin antiserum (Huang et al., 2006) (1:1,000).

Membranes were blocked for 1 h using Odyssey Blocking Buffer in TBS (Licor) prior to primary antibody incubation. All primary antibodies were incubated overnight at 4°C, and secondary antibodies at room temperature for 1 h. Anti-GAPDH (1:2,000, Abcam, ab8245) was used as a loading control. Alexa Fluor secondary antibodies (Invitrogen) were used at 1:10,000.

### Enrichment for Nuclear Proteins

Ventricles from PND1 WT and cMyBP-C^-/-^ mouse hearts were isolated and homogenate was fractionated to enrich for nuclear proteins using the NE-PER nuclear and cytoplasmic extraction kit (Thermo Scientific, cat #78833) according to manufacturer instructions. Total protein in nuclear fractions was quantified using a standard BCA protein assay (Thermo Fisher) using BSA protein standards. 25 μg of total protein homogenate was fractionated in 4–20% SDS-PAGE gels (Bio-Rad) and transferred overnight at 15 V in Tris–glycine buffer. Immunoblotting was performed for Zbtb16 (anti-Zbtb16; 1:1,000, Abcam, ab189849) and the nuclear loading control Histone 3 (anti-H3; 1:1,000, Cell Signaling cat# 9715), as described above for Xirp2.

### Statistical Analysis

Values are reported as means ± SE. For statistical analysis of microarray data, genes differentially expressed (up or down greater than twofold at *p* < 0.05) were identified using pairwise analysis of means and Student’s *t*-test (comparing WT to cMyBP-C^-/-^ and/or comparing PND1 to PND9) or using two-way ANOVA. For statistical analysis of qPCR and western blots, Student’s *t*-test was performed within each age group between genotypes, and statistical significance is reported at *p* < 0.05. All graphs were generated using GraphPad Prism and sample sizes are listed in figure legends.

## Results

We performed microarray analysis using RNA isolated from left ventricular free walls from cMyBP-C^-/-^ and WT mice at PND1 and PND9 to highlight differential RNA expression before and after the presentation of overt hypertrophy and dysfunction, and to uncover cell signaling that might influence the differential pathways leading to physiologic growth vs. pathologic hypertrophy. As the goal of this study was to reveal individual genes that may have profound influence on disease progression and have the strongest potential for physiological relevance, we restricted our dataset to genes that were up- or downregulated ≥2-fold, *p* < 0.05 relative to WT littermate controls for each selected comparison, unless noted otherwise. The microarray data were validated using qPCR performed on PND1 and PND9 hearts (see Supplementary Figure S1).

### Genes Involved in the Differential Development of Physiologic Versus Hypertrophic Growth

**FIGURE 1:**
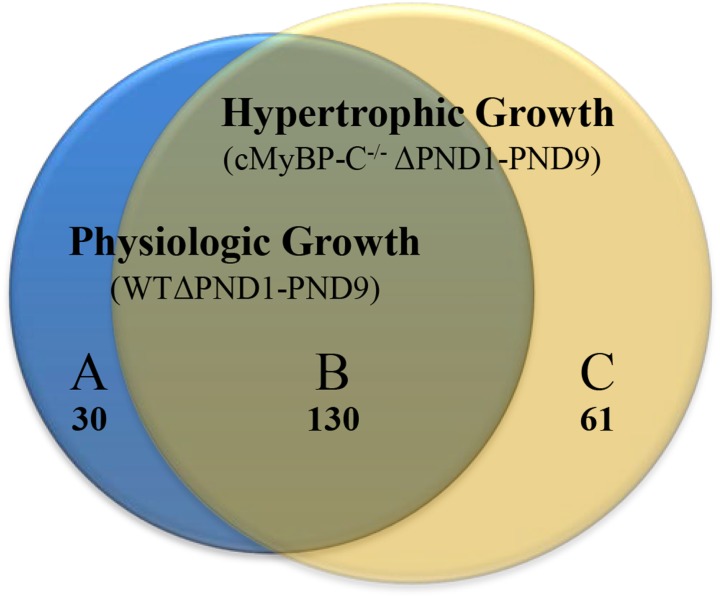
Venn diagram illustrating genes whose expression is changed ≥2-fold throughout normal, physiologic growth (WTΔPND1-PND9; blue circle), or hypertrophic growth (cMyBP-C^-/-^ΔPND1-PND9; yellow circle). Subsections of the Venn diagram include genes that are **(A)** exclusively regulated during physiologic growth, **(B)** regulated during both physiologic and hypertrophic growth, and **(C)** exclusively regulated during hypertrophic growth. Values listed indicate the number of genes that belong to each section. The genes which comprise each subsection of the Venn diagram are available in Supplementary Table S1 [physiologic growth **(A)**], Supplementary Table S2 [hypertrophic growth **(C)**], and Supplementary Table S3 [common to physiologic and pathophysiologic growth **(B)**].

Microarray analysis revealed that 30 genes were exclusively regulated during physiologic growth (WTΔPND1-PND9; Supplementary Table S1), 61 genes were exclusively regulated during hypertrophic growth (cMyBP-C^-/-^ΔPND1-PND9; Supplementary Table S2), and 130 genes were common to both physiologic and hypertrophic growth (Supplementary Table S3), represented by the intersection of two circles of a Venn diagram (Figure 1). The top-scoring Gene Ontology (GO) biological processes according to Toppcluster (Kaimal et al., 2010) (Cincinnati Children’s Biomedical Informatics) associated with each grouping in Figure 1 are listed in Table 1. From a list of dysregulated genes in a given model or condition, the Toppcluster software identifies GO biological processes to which the dysregulated genes belong. This is extremely useful for categorizing the myriad of gene changes. However, the identification of a GO biological process does NOT indicate the directional change of the gene’s dysregulation, nor consequently the impact on the process. For example, several identified GO processes unique to physiologic growth (WTΔPND1-PND9, section A of the Venn diagram) relate to hypertrophy, namely, “cardiac muscle hypertrophy” and “striated muscle hypertrophy” (Table 1). These processes are notably absent from the list for hypertrophic growth (cMyBP-C^-/-^ΔPND1-PND9, section C of the Venn diagram). Upon further investigation, we discovered that two genes which elicit the identification of the hypertrophic GO biological processes in physiologic growth are *Myh7* and *Nppa*. The expression of both of these genes goes *down*, as expected, during physiologic growth from PND1, when growth is high, to PND9, when growth has slowed. These genes are ascribed to “hypertrophic growth” pathways, regardless of the fact that their expression is regulated in the opposite direction to what is associated with hypertrophy. The absence of these GO biological processes from the analysis of hypertrophic growth reflects the unusual *maintenance* of gene expression of *Myh7* and *Nppa* from PND1 to PND9, which is reflective of activation of the hypertrophic pathway. Therefore, the utility in the identification of GO processes should be balanced with mindfulness of its limitations when interpreting results. It is of note that the top two GO biological processes associated with genes exclusively regulated during hypertrophic growth are both affiliated with extracellular matrix/structure. This suggests that extracellular signaling may be critical in the development of pathophysiologic hypertrophy. Additionally, the genes exclusively differentially expressed in physiologic growth (Supplementary Table S1) are of interest because they might be aberrantly non-responsive in cMyBP-C^-/-^ hearts.

**Table 1:** Top-scoring GO biological processes for genes regulated exclusively during physiologic (WT) or hypertrophic (cMyBP-C^-/-^) growth (ΔPND1-PND9), or genes regulated during all growth.

Physiologic growth (“A” on Venn diagram)	Physiologic and hypertrophic growth (“B” on Venn diagram)	Hypertrophic growth (“C” on Venn diagram)
Regulation of cell growth	Muscle organ development	EC matrix organization
Cell growth	Positive regulation of cardiac muscle	EC structure organization
Negative regulation of cell growth	Cardiovascular system development	Sclerotome development
Cardiac muscle hypertrophy	Circulatory system development	
Regulation of chemotaxis	Cardiac muscle tissue morphogenesis	
Striated muscle hypertrophy	Blood vessel morphogenesis	
Muscle hypertrophy	Muscle structure development	
Regulation of muscle system process	Muscle tissue morphogenesis	
Developmental cell growth	Vasculature development	
Muscle system process	Anatomical structure formation involved in morphogenesis	


*Unique biological processes regulated between physiologic (WTΔPND1-PND9) and hypertrophic (cMyBP-C^-/-^ΔPND1-PND9) growth. Genes within each category are listed in Supplementary Tables S1–S3.*

The 130 genes showing ≥2-fold changes during both physiologic and hypertrophic growth (Figure 1, section B of diagram; Supplementary Table S3) were all either up- or downregulated in the same direction in both types of growth. However, only two of these genes, *Xirp2* and *Bmp10 precursor*, also exhibited a ≥2-fold difference *between* WT and cMyBP-C^-/-^ hearts in the magnitude of that growth-related change in expression. Specifically, we calculated a fold-change ratio (FR) for each gene, which is the ratio of the fold change in expression during hypertrophic growth (cMyBP-C^-/-^ΔPND1-PND9) over the fold change in expression during physiologic growth (WTΔPND1-PND9). *Xirp2* and *Bmp10* were the only genes with an FR ≥ 2, highlighted in Table 2, an excerpt of Supplementary Table S3. Thus, these genes are regulated in both types of growth but show an exacerbated response in hypertrophic growth.

**Table 2:** Genes regulated ≥2-fold during both WT and cMyBP-C^-/-^ growth (ΔPND1-PND9), and that differ in magnitude of change by ≥2-fold between WT and cMyBP-C^-/-^ growth.

Gene name	Gene ID	Physiologic growth (WTΔPND1-PND9)		Pathophysiologic growth (cMyBP-C ΔPND1-PND9)		Fold-change ratio (cMyBP-C^-/-^/WT)
				
		Fold-change	Direction	Fold-change	Direction	
Xin actin-binding repeat containing 2	Xirp2	3.06	Up	6.13	Up	2.0
Bone morphogenetic protein 10 precursor	Bmp10	2.06	Down	4.09	Down	2.0


*Genes listed in this table are contained within section B of the Venn diagram (Figure 1). Fold-change ratio is the ratio of fold-change expression during cMyBP-C^-/-^ growth (fifth column) divided by the fold-change expression during WT growth (third column). A fold-change ratio greater than 1 indicates a greater degree of change (up or down) in the cMyBP-C^-/-^ compared to WT hearts. Both genes are implicated in “circulatory system development” and “cardiovascular system development” by Toppcluster. Data in this table are an excerpt from Supplementary Table S3, highlighting only genes with a fold-change ratio of ≥2.*

### Identification of Growth-Related Genes With Early Dysregulation, Prior to Hypertrophy

To further highlight potential important mediators in the hypertrophic pathway, we probed the data set of genes that were changed ≥2-fold during hypertrophic growth (Figure 1; cMyBP-C^-/-^ΔPND1-PND9, yellow circle) for any genes that were also dysregulated ≥2-fold prior to hypertrophy, at PND1, in cMyBP-C^-/-^ vs. WT left ventricles. At PND1, the hearts of the cMyBP-C^-/-^ pups are virtually indistinguishable from WT littermates, both morphologically and functionally (de Lange et al., 2013; Farrell et al., 2017). This gene subset might include growth-related genes with an early, pre-hypertrophic response that may be involved in phenotype progression and may thus represent potential therapeutic targets. Only six genes fit these criteria: *Xirp2, Aqp7, Zbtb16, Rxfp1, Ucp3*, and *Olfr608* (Table 3). *Xirp2, Aqp7*, and *Olfr608* are regulated during both physiologic and pathophysiologic growth, and thus are represented within section B of the Venn diagram in Figure 1, and *Zbtb16, Rxfp1, Ucp3* are differentially regulated during hypertrophic but not physiologic growth (section C of the Venn diagram). Because *Aqp7, Zbtb16, Rxfp1*, and *Ucp3* are regulated at PND1 in the opposite directions to that of hypertrophic growth, we did not initially further pursue these genes as potential early response genes of hypertrophy. *Olfr608* encodes for a protein in the olfactory receptor superfamily, which recently has been shown to include some members with expression in the heart (Flegel et al., 2013), one of which is reported to affect cardiac function (Jovancevic et al., 2017). However, to the best of our knowledge, the function of *Olfr608* in cardiomyocytes is unknown. Xirp2 is a sarcomere-related protein whose expression is confined to striated muscle with reported localization to intercalated discs (Wang et al., 2010; Wang Q. et al., 2012), costameres (Huang et al., 2006), and/or z-discs (Huang et al., 2006; Eulitz et al., 2013). Xirp2 has been shown to be upregulated in some cardiomyopathies (Jung-Ching Lin et al., 2005; Duka et al., 2006; Wang et al., 2014) and mice engineered to express reduced levels of Xirp2 are partly protected from angiotensin-II-induced cardiomyopathic remodeling (McCalmon et al., 2010). In the current study, *Xirp2* was the most dysregulated gene in our cMyBP-C^-/-^ hearts at PND1, with 2.87-fold elevated RNA expression vs. WT (Table 4), and the fourth most differentially regulated gene at PND9, when overt hypertrophy is evident (Supplementary Table S4). Given that *Xirp2* was also one of only two genes to have ≥ 2-fold differential growth regulation in hypertrophic growth relative to physiologic growth (illustrated by FR ≥ 2, Table 2), as mentioned previously, we chose to pursue *Xirp2* as a gene of interest for its potential as an early-response gene mediating hypertrophic signaling prior to the onset of hypertrophy.

**Table 3:** Potential hypertrophy early responder genes.

Gene name	Gene ID
Xin actin-binding repeat containing 2^∗^	Xirp2
Aquaporin 7	Aqp7
Olfactory receptor 608^∗^	Olfr608
Zinc finger and BTB domain containing 16	Zbtb16
Relaxin/insulin-like family peptide receptor 1	Rxfp1
Uncoupling protein 3 (mitochondrial, proton carrier)	Ucp3


*All genes listed were dysregulated in the cMyBP-C^-/-^ vs. WT at both PND1 and during growth (ΔPND1-PND9). ^∗^Genes that were differentially regulated in the same direction for both the PND1 WT vs. cMyBP-C^-/-^ analysis AND for analysis of normal and hypertrophic growth.*

**Table 4:** Genes upregulated or downregulated ≥2-fold at PND1 in cMyBP-C^-/-^ vs. WT.

Gene name	Gene ID	Ratio	Direction	*p*-value	Gene identifier
Xin actin-binding repeat containing 2	Xirp2	2.87	Up	0.0061	NM_001024618
Zinc finger and BTB domain containing 16	Zbtb16	2.40	Up	0.0228	NM_001033324
Neurotensin/neuromedin N	Nts	2.20	Up	0.0378	NM_024435
Relaxin/insulin-like family peptide receptor 1	Rxfp1	2.03	Up	0.0398	NM_212452
Myosin-binding protein C, cardiac	Mybpc3	17.50	Down	0.0000	NM_008653
Carbonic anhydrase 4	Ca4	2.42	Down	0.0012	NM_007607
Uncoupling protein 3 (mitochondrial, proton carrier)	Ucp3	2.26	Down	0.0312	NM_009464
Rho GTPase activating protein 36	Arhgap36	2.20	Down	0.0006	NM_001081123
Olfactory receptor 608	Olfr608	2.10	Down	0.0053	NM_146756
Small nuclear ribonucleoprotein N	Snrpn	2.05	Down	0.0106	NM_013670
Aquaporin 7	Aqp7	2.05	Down	0.0114	NM_007473


### Elevated Xirp2 Gene and Protein Expression in cMyBP-C^-/-^ Hearts From Birth to Hypertrophy

**FIGURE 2:**
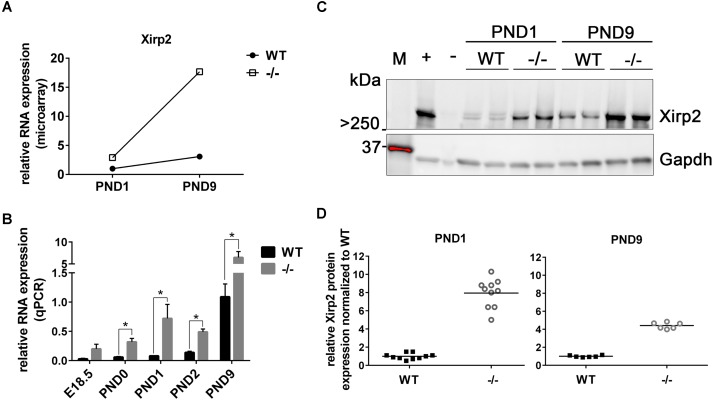
Sustained upregulation of *Xirp2* mRNA and Xirp2 protein in cMyBP-C^-/-^ vs. WT hearts from birth through PND9. **(A)** Microarray analysis of relative *Xirp2* mRNA expression from left ventricular free wall of WT and cMyBP-C^-/-^ hearts at PND1 and PND9 (*n* = 7). **(B)** mRNA expression of *Xirp2* from WT and cMyBP-C^-/-^ hearts relative to the average of housekeeping genes, *Gapdh* and *β-actin*, at E18.5, PND0, PND1, PND2, and PND9 using qPCR. Means ± SE are reported, ^∗^*p* < 0.05 (*n* = 5). **(C)** Representative western blot of Xirp2 in WT and cMyBP-C^-/-^ (–/–) ventricular homogenate from PND1 and PND9, with tongue homogenate and spleen homogenate loaded as positive (+) and negative (–) controls, respectively. M; molecular weight markers. **(D)** Relative Xirp2 protein expression normalized to WT quantified from Western blots. Horizontal lines represent means, ^∗^*p* < 0.05 [*n* = 10 (PND1); 6 (PND9)].

As stated, microarray analysis revealed elevated *Xirp2* gene expression in cMyBP-C^-/-^ left ventricles relative to WT at PND1 and PND9 (Table 4 and Figure 2A). Using qPCR, we confirmed that *Xirp2* expression is higher than WT at these day points and further assessed time points of embryonic day 18.5 (E18.5), PND0 (day of birth), and PND2, to better understand the time course of *Xirp2* gene expression (Figure 2B). *Xirp2* expression was found to be elevated with respect to WT at each time point from PND0 through PND9, and trended toward being increased, although not significantly, at E18.5. Protein levels of Xirp2 were correspondingly increased at the assessed time points of PND1 (7.9 ± 1.49-fold increase over WT) and PND9 (4.4 ± 0.35-fold increase over WT; Figures 2C,D).

### Increased Localization of Xirp2 to End Terminals of Cardiomyocytes in cMyBP-C^-/-^ Hearts Prior to Hypertrophy

**FIGURE 3:**
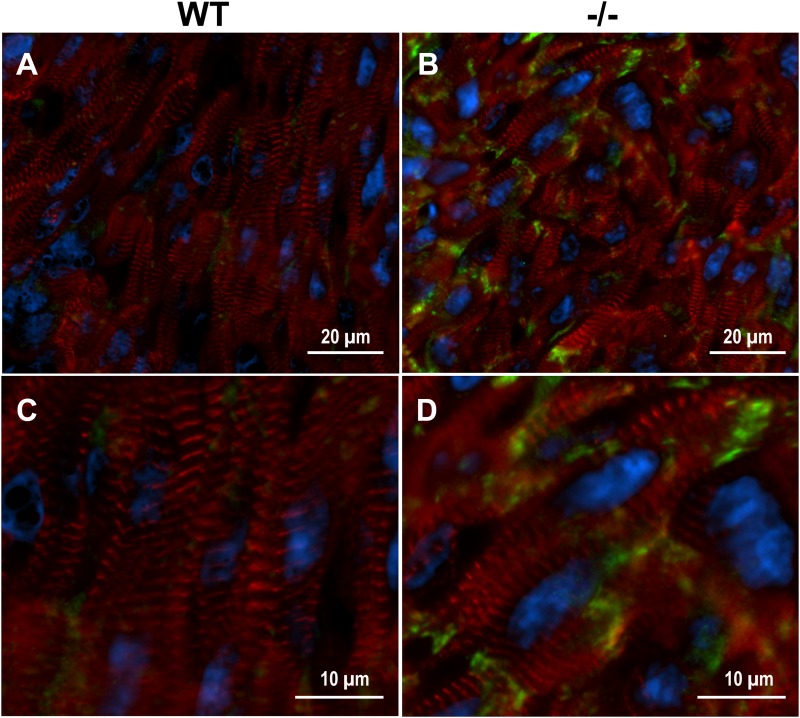
Enhanced localization of Xirp2 to cell-to-cell junctions in cMyBP-C^-/-^ mouse hearts at PND1. Immunohistochemistry showing α-actinin (red), nuclei (DAPI, blue), and Xirp2 (green) in WT **(A,C)** and cMyBP-C^-/-^ **(B,D)** mouse heart cryosections at PND1. Magnified images are shown in **(C,D)**.

Xirp2 is required for relocating intercalated disc proteins from the lateral edges of cardiomyocytes, where they reside until after birth, to their final destination at the end termini of cardiomyocytes (Wang et al., 2010, 2014; Wang Q. et al., 2012). Mice lacking Xirp2 fail to develop mature intercalated discs, exhibit cardiac myopathy and diastolic dysfunction, and die prior to weaning (Wang et al., 2010). Immunolabeling of cMyBP-C^-/-^ and WT PND1 cryogenic heart sections revealed intensified protein localization of Xirp2 to end-terminals of cMyBP-C^-/-^ cardiomyocytes compared to WT (Figure 3). This is consistent with the reporting of Xirp2 localization at intercalated discs of mature cardiomyocytes (Wang et al., 2010; Wang Q. et al., 2012).

### Early Upregulation of RNA and Nuclear Localized Protein of the Transcription Factor Zbtb16 in cMyBP-C^-/-^ Hearts

**FIGURE 4:**
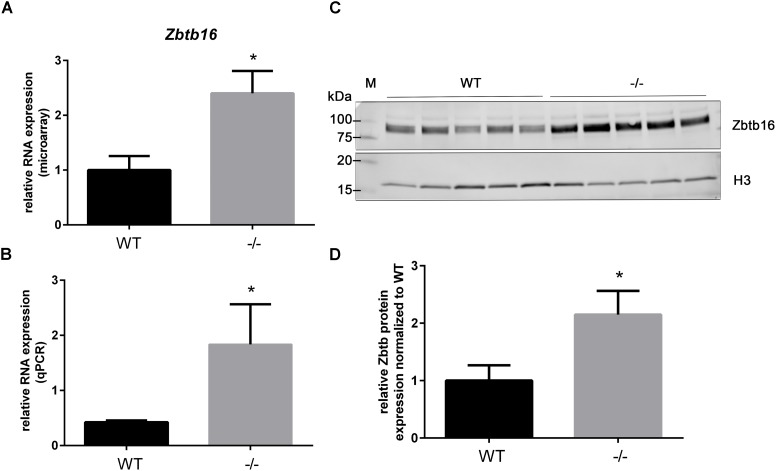
Upregulation of *Zbtb16* mRNA and nuclear protein in cMyBP-C^-/-^ vs. WT hearts at PND1. **(A)** Microarray analysis of relative *Zbtb16* mRNA expression from left ventricular free wall of WT and cMyBP-C^-/-^ hearts at PND1 (*n* = 7 per genotype). **(B)** mRNA expression of *Zbtb16* from WT and cMyBP-C^-/-^ hearts relative to the average of housekeeping genes, *Gapdh* and *β-actin*, at PND1 using qPCR. Means ± SE are reported, ^∗^*p* < 0.05 [*n* = 8 (WT), 5 (–/–)]. **(C)** Western blot of Zbtb16 in WT and cMyBP-C^-/-^ (-/-) ventricular homogenate from PND1 with Histone 3 (H3) run as a nuclear loading control. M; molecular weight markers. **(D)** Relative Zbtb16 nuclear protein expression normalized to WT quantified from Western blots. Means ± SE are graphed, ^∗^*p* < 0.05 (*n* = 5 per genotype).

As previously shown in Table 3, the gene Zinc finger and BTB domain containing 16 (*Zbtb16*; aka *Plzf*), is categorized as a potential early responder gene because its expression is significantly higher than WT at PND1 (2.4-fold, Table 4 and Figure 4A) and because it shows growth-related changes in expression. However, Zbtb16 RNA in cMyBP-C^-/-^ hearts returns to near WT levels by PND9. Despite *Zbtb16*’s unsustained upregulation, its robust early elevation at PND1 combined with its role as a transcription factor prompted us to further evaluate its expression. We hypothesized that a transient, but marked upregulation of a transcription factor may indeed have lasting effects on the proteome. Notably, after *Xirp2, Zbtb16* was the second most dysregulated gene at PND1 in the cMyBP-C^-/-^ hearts (Table 4). Thus, we first verified upregulation of RNA expression at PND1 through qPCR (4.33 ± 1.72 fold vs. WT; Figure 4B). Next, we determined protein expression through western blotting of Zbtb16 in a nuclear-enriched fraction of WT and cMyBP-C^-/-^ hearts at PND1 and similarly found increased nuclear Zbtb16 protein expression relative to the nuclear marker histone 3 (H3) (2.15 ± 0.41 fold vs. WT; Figures 4C,D).

### Altered Gene Expression in Mechanosensing Regions of Cardiomyocyte in cMyBP-C^-/-^ Hearts

**FIGURE 5:**
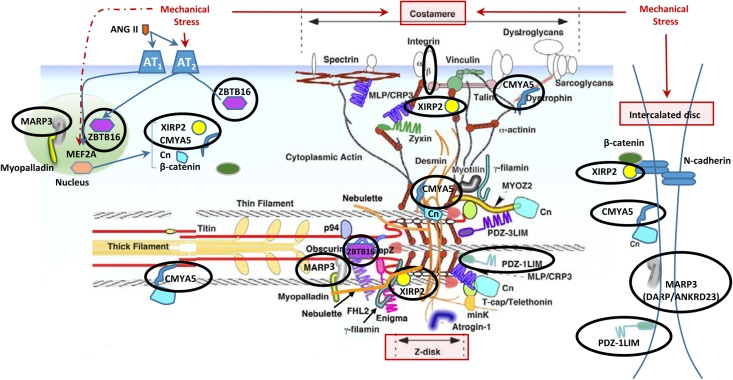
Altered gene expression in cMyBP-C^-/-^ hearts in cardiomyocyte mechanosensing regions of the intercalated discs, z-disks, and costameres. Circled are proteins whose genes were found to be dysregulated ≥1.5-fold in the cMyBP-C^-/-^ mouse by microarray. Note that some protein products are repeated in the figure as they have been shown to localize to multiple areas. This figure is modified from Hoshijima (2006) and printed with permission. Xirp2, xin actin-binding repeat containing 2; Zbtb16, zinc finger and BTB domain containing 16; Marp3, ankyrin repeat domain 23/Ankrd23/DARP; PDZ-1LIM, PDZ, and LIM domain 3/Pdlim3; Cmya5, cardiomyopathy-associated 5/Myopryn; Cn, calcineurin; MYOZ2, myozenin 2; FHL2, four and a half LIM domains 2; MinK, MINK1/misshapen-like kinase 1; MLP/CRP3, cysteine and glycine-rich protein 3/CSRP3/muscle LIM protein; Ang II, angiotensin II; p94, calpain 3/CAPN3; PDZ-3LIM, enigma gene family; Mef2a, myocyte enhancer factor 2A.

**FIGURE 6:**
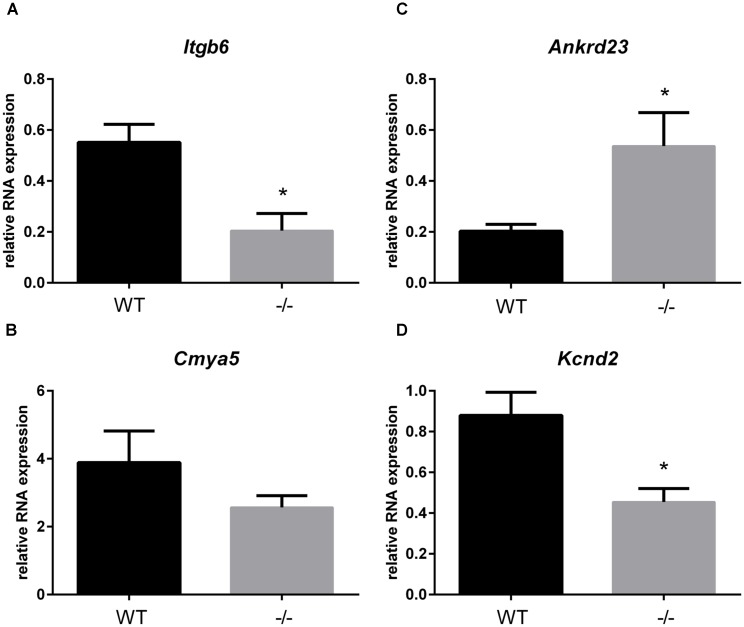
RNA expression of genes in mechanosensing regions of the intercalated discs, z-disks, and costameres in cMyBP-C^-/-^ vs. WT hearts at PND1. mRNA expression of *Itgb6* **(A)**, *Cmya5* **(B)**, *Andkrd23* **(C)**, and *Kcnd2* **(D)** from WT and cMyBP-C^-/-^ hearts relative to the average of housekeeping genes, *Gapdh* and *β-actin*, at PND1 using qPCR. Means ± SE are reported, ^∗^*p* < 0.05 [*n* = 7 (WT), 6 (-/-)].

In addition to the localization of Xirp2 to junctions of cardiomyocytes that we (Figure 3) and others have shown (Wang et al., 2010; Wang Q. et al., 2012), localization to costameres and/or z-discs has also been reported (Huang et al., 2006; Eulitz et al., 2013). Indeed, several known binding partners of Xirp2 [e.g., γ-filamin (Kley et al., 2013), nebulette (Eulitz et al., 2013), and α-actinin (Huang et al., 2018)] reside in costameres and/or z-discs. This localization of Xirp2 places this essential protein in known mechanosensing regions of the cardiomyocyte, suggesting that Xirp2 might play a role in sensing and/or responding to mechanical stress placed on the cardiomyocytes due to the primary sarcomeric mutation in cMyBP-C. To further probe this possibility, we examined the microarray data to determine whether the genes of other proteins located in these mechanosensing regions were also dysregulated in the cMyBP-C^-/-^ hearts. For this analysis, we lowered the fold change threshold to 1.5 (*p* < 0.05) in order to highlight genes that may be more modestly altered, but together might indicate a common pathway of dysregulation leading to hypertrophy. Figure 5 diagrams the purported locations of Xirp2 in the mechanosensing regions of the intercalated disc, costamere, and z-discs, and highlights those proteins whose genes were found to be dysregulated in cMyBP-C^-/-^ left ventricles. Zbtb16 is also depicted in Figure 5 because the translocation of this upregulated transcription factor to the nucleus can be induced by mechanical stress. Supplementary Table S5 lists the specific dysregulation of the genes encoded by proteins circled in Figure 5. To further validate the early dysregulation reported in the microarray of genes in mechanosensing regions, qPCR was performed at PND1 on *Ankrd23, Itgb6*, and *Cmya5* (Figure 6A–C). Zbtb16 (Figure 4) and Xirp2 (Figure 2) were previously verified. Although expression of *Cmya5* was not statistically different from WT in qPCR, the direction and magnitude of change was comparable to the microarray (Figure 6).

### Early Alterations in Gene Expression of Potassium Channels in cMyBP-C^-/-^ Hearts

A recent study using the cMyBP-C^-/-^ mouse model focused on the remodeling of action potential repolarization. Based on their data, the authors suggested that remodeled potassium (K^+^) currents in adult cMyBP-C^-/-^ hearts are the result of altered expression of K^+^ channels and contribute to the incidence of SCD in HCM (Toib et al., 2017). Thus, we probed our microarray data and found that many K^+^ channel genes as well as one gene encoding a K^+^ channel interacting protein, were dysregulated either at PND1 or PND9, or both, at a fold change threshold set at 1.5 (Table 5). These data corroborate the report that K^+^ channel dysregulation is present in HCM, and further suggest that the dysregulation is not a product of a hypertrophic heart, but precedes it. We further investigated this early, pre-hypertrophic K^+^ channel dysregulation by performing qPCR on the K^+^ channel subunit K_v_4.2 (*Kcnd2*) at PND1, as *Kcnd2* was the most robustly downregulated gene reported in the adult cMyBP-C^-/-^ hearts according to Toib et al. (2017). qPCR supported the microarray results, showing a 2.2 ± 0.44-fold lower RNA expression of *Kcnd2* in cMyBP-C^-/-^ hearts compared to WT at PND1 (Figure 6D).

**Table 5:** Genes encoding potassium channels or their interacting proteins, regulated ≥1.5 fold in cMyBP-C^-/-^ hearts at PND1 or PND9.

Gene name	Gene ID	PND1		PND9	
			
		Ratio	Direction	Ratio	Direction
Potassium voltage-gated channel subfamily E regulatory subunit 3	Kcne3	1.71	Up	1.96	Up
Hyperpolarization activated cyclic nucleotide gated potassium channel 1	Hcn1			1.61	Up
Potassium voltage-gated channel subfamily D member 2	Kcnd2	1.59	Down	2.93	Down
Potassium voltage-gated channel subfamily B member 1	Kcnb1	1.60	Down		
Potassium calcium-activated channel subfamily N member 2	Kcnn2	1.61	Down		
Potassium voltage-gated channel subfamily J member 3	Kcnj3	1.74	Down	2.08	Down
Potassium voltage-gated channel interacting protein 2	Kcnip2			1.52	Down
ATPase Na^+^/K^+^ transporting subunit alpha 2	Atp1a2			1.87	Down


*Ratio and direction are relative to WT at the same age.*

## Discussion

Through microarray analysis of an HCM mouse model in which cMyBP-C is genetically ablated, we revealed a gene of interest, *Xirp2*, which is upregulated almost threefold in the cMyBP-C^-/-^ heart starting prior to any overt hypertrophy or signs of dysfunction (Table 4 and Figure 2A). *Xirp2*, also known as cardiomyopathy-associated protein 3 (*Cmya3*), was first identified in cardiac tissue of humans with cardiomyopathy (Duka et al., 2006) and has been shown to be upregulated in other forms of cardiac disease, including pressure-overload hypertrophy (Wang Q. et al., 2012) and during angiotensin-II remodeling (Duka et al., 2006; McCalmon et al., 2010), yet it is decreased in heart failure and idiopathic dilated cardiomyopathy (Wang et al., 2014). Additionally, *Xirp2* has been implicated as a genetic modifier in a childhood case of severe dilated cardiomyopathy (Long et al., 2015). In this case, compound heterozygous truncation mutations in *Xirp2* were found in conjunction with a troponin T mutation. Interestingly, the heart demonstrated severe, biventricular hypertrophy, along with abnormal and mislocalized intercalated discs. Xirp2 is required for the formation and proper localization of mature intercalated discs, and mice completely deficient in Xirp2 exhibit diastolic dysfunction and die prior to weaning age (Wang et al., 2010, 2014; Wang Q. et al., 2012). Underexpression of Xirp2 leads to cardiac hypertrophy in mice, yet mitigates the cardiac fibrosis and apoptosis induced by angiotensin II (McCalmon et al., 2010), suggesting that tight control of Xirp2 protein levels is required for a healthy myocardium. *Xirp2* appears to be under the direct control of the transcription factor Mef2a, which binds to the *Xirp2* promoter and regulates transcription of Xirp2 (Huang et al., 2006; Wang Q. et al., 2012). The *Xirp2* gene also harbors a nuclear localization sequence, a nuclear export sequence, and a DNA-binding region (Wang Q. et al., 2012). Although their functions remain unknown, these regions of DNA suggest that Xirp2 may have the capacity to regulate transcription.

*Xirp2* was not only the most dysregulated gene at PND1, but maintained this differential upregulation with respect to WT through PND9, although a growth-related increase in *Xirp2* was observed in both physiologic and hypertrophic growth (Table 2 and Figure 2A). We show that the upregulation in *Xirp2* gene expression translates to an increase in protein product in cMyBP-C^-/-^ over WT of almost eightfold at PND1 and 4.4-fold at PND9 (Figures 2C,D), with intensified localization at the intercalated disc (Figure 3), a previously identified subcellular location of the protein and a region capable of sensing mechanical stress (Hoshijima, 2006; Lyon et al., 2015). It is of note that we did not observe identifiable labeling of Xirp2 at other purported sites of localization, namely, at costameres and/or z-discs in these neonatal mouse cardiomyocytes. It is possible that with different potential binding partners in the multiple locations, different epitopes are exposed, allowing for certain antibodies to bind in one location and not another. Furthermore, development/condition may also play a role in Xirp2 localization. Indeed, immunohistochemistry using neonatal mouse myocardium shows localization of Xirp2 to intercalated discs (Wang et al., 2010) while in adult hearts it is found at the z-disc/costamere (Huang et al., 2006).

Further examination of the microarray revealed four additional genes (*Cmya5, Darp/Ankrd23, Pdz-1lim/Pdlim3*, and *Itgb6*) which were dysregulated ≥1.5-fold in cMyBP-C^-/-^ hearts and whose protein products reside in the mechanosensing regions of the costamere, z-disc, and/or intercalated disc (Figure 5 and Supplementary Table S5). Additionally, *Zbtb16*, which was the second most dysregulated gene at PND1 according to microarray analysis (Table 4), showed mRNA upregulation relative to WT using qPCR, and upregulated protein expression in the nucleus (Figure 4). *Zbtb16* is a transcription factor that translocates to the nucleus upon stimulation of the angiotensin II type 2 receptor (Senbonmatsu et al., 2003), which itself can be directly activated by mechanical stress (Zou et al., 2004). Furthermore, *Zbtb16* is known to regulate GATA4 transcription (Wang N. et al., 2012) and has a proposed role in cardiac hypertrophy (Senbonmatsu et al., 2003; Wang N. et al., 2012). This early transcriptional activation in the cMyBP-C^-/-^ hearts suggests that the contractile changes related to absence of cMyBP-C induce a mechanical perturbation at the level of the sarcomere that may be transduced to the intercalated disc, z-disc, and/or costamere, triggering the observed alterations in gene expression in these regions. While further studies are required to validate this hypothesis, the idea that these regions are involved in mechanical stress sensing is well-reported (Hoshijima, 2006; Cox et al., 2008; Frank and Frey, 2011; Granados-Riveron and Brook, 2012; Lyon et al., 2015). Intercalated discs have also been shown to remodel during cardiac stress induced by transverse aortic constriction-mediated pressure overload (Kebir et al., 2016).

This study also indicates additional genes and biological processes that are dysregulated throughout hypertrophic growth and/or prior to hypertrophy. The identification of extracellular matrix organization and extracellular structure organization as the top two GO biological processes resulting from genes exclusively dysregulated in hypertrophic growth (Table 1) implicates early extracellular matrix remodeling as a primary response to the loss of cMyBP-C. The structural continuity between the extracellular matrix and intracellular cytoskeletal elements through costameric and intercalated disc proteins further suggests a role for mechanical stress in triggering the HCM phenotype in cMyBP-C^-/-^. One limitation of the study is that microarray, the technique available to us at the time of the study, is less sensitive than newer methods such as RNA Seq. Although the lower sensitivity of microarray does not minimize our ability to identify robust changes in gene expression, there may be pathway dysregulation that microarray analysis fails to detect.

Myosin-binding protein C has three main isoforms, slow skeletal (sMyBP-C; *Mybpc1*), fast skeletal (fMyBP-C; *Mybpc2*), and cardiac (cMyBP-C; *Mybpc3*), each encoded by different genes, as listed. Although the cardiac isoform, cMyBP-C, is the only one found in healthy hearts, fMyBP-C protein has been shown to be expressed in a dilated cardiomyopathy-induced heart failure mouse model [cMyBP-C^(t/t)^] (Lin et al., 2013). Similarly, we observed an upregulation of *Mybpc2* RNA expression in the cMyBP-C^-/-^ ventricles relative to WT, both in the microarray and in targeted qPCR (Supplementary Figure S1). Given that expression was elevated at PND1 as well as at PND9, *Mybpc2* upregulation was not a result of hypertrophy or cardiac dysfunction, as it preceded both. WT hearts also showed low levels of RNA expression; however, protein levels were not determined in hearts of either genotype. It is therefore not known whether fMyBP-C protein is produced and/or incorporated into the sarcomere of the cMyBP-C^-/-^ cardiomyocytes. Future studies will be aimed at addressing this question.

The role of the intercalated disc and Xirp2 in the etiology of HCM may extend beyond hypertrophic signaling to include arrhythmogenesis, an important hypertrophy-independent manifestation of HCM. The intercalated disc contains many ion channels and proteins known to regulate the production and localization of ion channels involved in formation of the action potential and conduction of the electrical signal (Chan et al., 2011; Swope et al., 2012; Wang and Lin, 2013; Vermij et al., 2017). Mutations in intercalated disc ion channels and in non-conducting intercalated disc proteins are known to result in arrhythmias, including SCD (Swope et al., 2012; Wang et al., 2014; Vermij et al., 2017). Recently, mutations in *Xirp2* itself were shown to be responsible for some cases of arrhythmias in sudden unexplained nocturnal cardiac death (Huang et al., 2018). Furthermore, a recent study using the same cMyBP-C^-/-^ mouse model as used in our study, suggests that remodeled K^+^ currents in adult cMyBP-C^-/-^ hearts are the result of altered expression of K^+^ channels and contribute to the incidence of SCD in HCM (Toib et al., 2017). Our microarray data provide additional support for this hypothesis as there was important dysregulation of several K^+^ channels at PND1 and/or PND9 in the cMyBP-C^-/-^ hearts. Interestingly, the K^+^ channel gene shown to be most downregulated in the adult cMyBP-C^-/-^ heart, *Kcnd2*, coding for K_v_4.2, also localizes to intercalated discs (as well as peripheral sarcolemma) along with *Kcnb1*/K_v_2.1 (Barry et al., 1995; Takeuchi et al., 2000). Our data show downregulation of K_v_4.2 mRNA (at PND1, prior to hypertrophic remodeling and cardiac dysfunction, and at PND9) (Table 5 and Figure 6D). This finding not only supports the work of Toib et al., but additionally suggests that the onset of alterations of several potassium channels, including *Kcnd2*/K_v_4.2, is not secondary to the hypertrophic remodeling of the heart, but may substantially precede it, similar to *Xirp2*. Indeed, SCD can be unrelated to the degree of hypertrophic remodeling and can occur in the complete absence of hypertrophy (Moolman et al., 1997; Christiaans et al., 2009). Xirp1, encoded by the *Xirp1* gene, is a xin protein related to Xirp2 that also localizes to the intercalated discs. Interestingly, Xirp1 has been shown to directly influence the activity of K_v_4.2 (Chan et al., 2011; Wang Q. et al., 2012), which can prolong action potentials and induce arrhythmias, further supporting the postulate that the proteins in the intercalated disc play a role in modulating the electrical activity of the cardiomyocyte and may contribute to HCM-related arrhythmogenesis.

The intercalated disc is a region where mechanical sensing and transduction, cell signaling (e.g., hypertrophic), and electrical signaling converge. It is also intimately and directly connected to the active sarcomere at the transitional junction of the intercalated disc (Bennett et al., 2006; Vermij et al., 2017). The intercalated disc may well be a control tower responding directly to the mechanical stress resulting from the basic HCM-causing sarcomeric mutation through the transitional junction. The early, pre-hypertrophic upregulation of Xirp2 at the intercalated disc suggests that it may occupy a unique position in the cascade of cellular responses that result in the morphological and electrical phenotypes of HCM. It is not known whether Xirp2 plays a role in the early postnatal increase in cell cycling (Jiang et al., 2015; Farrell et al., 2017; Nixon et al., 2017) and/or cardiomyocyte proliferation (Jiang et al., 2015; Farrell et al., 2017) that we and others have observed in cMyBP-C-disrupted models, or if its role is entirely independent. We hypothesize that the homeostasis of Xirp2, and potentially its temporal expression, are important for normal cardiac function and that either elimination or overexpression of the protein are disruptive. It is also possible that Xirp2 is involved in an initial compensatory response to cardiac stress that triggers cell signaling pathways.

The data presented here support a need for further studies into the roles of *Xirp2, Zbtb16*, and *Kcnd2* in HCM. Manipulation of *Xirp2* expression in cardiomyocytes would be an important next step in delineating its role in HCM signaling. The large transcript size (11,955 bp encoding 3,283 amino acids) imposes significant challenges for manipulating gene expression. Although viral-mediated knockdown of *Xirp2* in cultured cardiomyocytes would be feasible, the failure of hypertrophic remodeling in cultured neonatal mouse cardiomyocytes makes assessment of the impact of gene manipulation difficult. Adenoviral-associated viruses (AAVs) injected into whole animals have become very effective in tissue-specific gene targeting; however, as the onset of *Xirp2* dysregulation begins in the cMyBP-C^-/-^ heart on or before the day of birth, the timing required for effective targeting may be unattainable. Future studies in human induced pluripotent stem cell cardiomyocytes may prove the most revealing, as they demonstrate the capacity to hypertrophy in culture (Eschenhagen et al., 2015; Kamdar et al., 2015; Tzatzalos et al., 2016), and have the additional advantage of reflecting an entirely human genetic context.

## Conclusion

Microarray analysis of cMyBP-C^-/-^ left ventricles both prior to and after the onset of the HCM phenotype generated a transcriptome profile comparing normal physiologic vs. hypertrophic growth. cMyBP-C^-/-^ induces hypertrophic growth that uniquely dysregulates pathways associated with the organization of extracellular matrix and extracellular structure. Microarray and qPCR evidence also highlighted the pre-hypertrophic dysregulation of potassium channel gene expression, including that of *Kcnd2*, encoding K_v_4.2, which has been recently linked to arrhythmias in HCM (Toib et al., 2017). Our data further identified the early dysregulation of *Xirp2*, a cardiomyopathy-associated gene whose protein product localizes to mechanosensing regions of the sarcomere, as well as dysregulation of a transcription factor, *Zbtb16*. Upregulation of *Xirp2* and *Zbtb16* is coincident with the dysregulation of several genes in the cMyBP-C^-/-^ mice that are involved in intercalated disc formation and/or mechanosensing. Future work will investigate the relationships between mechanical stress caused by sarcomere mutations, early dysregulation of the mechanosensing pathways, and the development of the hypertrophic and arrhythmogenic HCM phenotype.

## Author Contributions

EF, AG, WdL, and JR were involved in study design. EF, AA, AG, FN, WdL, and JR were involved in interpretation of data and writing. EF, AA, AG, FN, and WdL were involved in data collection and analysis. All authors contributed to editing.

## Conflict of Interest Statement

The authors declare that the research was conducted in the absence of any commercial or financial relationships that could be construed as a potential conflict of interest.

## References

[B1] Alfares A. A. Kelly M. A. McDermott G. Funke B. H. Lebo M. S. Baxter S. B. (2015). Results of clinical genetic testing of 2,912 probands with hypertrophic cardiomyopathy: expanded panels offer limited additional sensitivity. *Genet. Med.* 17 880–888. 10.1038/gim.2014.205 25611685

[B2] Barry D. M. Trimmer J. S. Merlie J. P. Nerbonne J. M. (1995). Differential expression of voltage-gated K + channel subunits in adult rat heart. Relation to functional K + channels? *Circ. Res.* 77 361–369. 7614722 10.1161/01.res.77.2.361

[B3] Bennett P. M. Maggs A. M. Baines A. J. Pinder J. C. (2006). The transitional junction: a new functional subcellular domain at the intercalated disc. *Mol. Biol. Cell.* 17 2091–2100. 10.1091/mbc.e05-12-1109 16481394 PMC1415289

[B4] Chan F. C. Cheng C. P. Wu K. H. Chen Y. C. Hsu C. H. Gustafson-Wagner E. A. (2011). Intercalated disc-associated protein, mXin-alpha, influences surface expression of ITO currents in ventricular myocytes. *Front. Biosci.* 3 1425–1442. 21622147 10.2741/e344 PMC3278966

[B5] Christiaans I. Lekanne dit Deprez R. H. van Langen I. M. Wilde A. A. (2009). Ventricular fibrillation in MYH7-related hypertrophic cardiomyopathy before onset of ventricular hypertrophy. *Heart Rhythm.* 6 1366–1369. 10.1016/j.hrthm.2009.04.029 19539541

[B6] Cox L. Umans L. Cornelis F. Huylebroeck D. Zwijsen A. (2008). A broken heart: a stretch too far: an overview of mouse models with mutations in stretch-sensor components. *Int. J. Cardiol.* 131 33–44. 10.1016/j.ijcard.2008.06.049 18715658

[B7] Davies M. J. McKenna W. J. (1995). Hypertrophic cardiomyopathy–pathology and pathogenesis. *Histopathology* 26 493–500. 10.1111/j.1365-2559.1995.tb00267.x 7665141

[B8] de Lange W. J. Grimes A. C. Hegge L. F. Ralphe J. C. (2013). Ablation of cardiac myosin-binding protein-C accelerates contractile kinetics in engineered cardiac tissue. *J. Gen. Physiol.* 141 73–84. 10.1085/jgp.201210837 23277475 PMC3536521

[B9] Duka A. Schwartz F. Duka I. Johns C. Melista E. Gavras I. (2006). novel gene (Cmya3) induced in the heart by angiotensin II-dependent but not salt-dependent hypertension in mice. *Am. J. Hypertens.* 19 275–281. 10.1016/j.amjhyper.2005.08.017 16500513

[B10] Eschenhagen T. Mummery C. Knollmann B. C. (2015). Modelling sarcomeric cardiomyopathies in the dish: from human heart samples to iPSC cardiomyocytes. *Cardiovasc. Res.* 105 424–438. 10.1093/cvr/cvv017 25618410 PMC4349163

[B11] Eulitz S. Sauer F. Pelissier M. C. Boisguerin P. Molt S. Schuld J. (2013). Identification of Xin-repeat proteins as novel ligands of the SH3 domains of nebulin and nebulette and analysis of their interaction during myofibril formation and remodeling. *Mol. Biol. Cell.* 24 3215–3226. 10.1091/mbc.E13-04-0202 23985323 PMC3810769

[B12] Fananapazir L. Epstein N. D. (1994). Genotype-phenotype correlations in hypertrophic cardiomyopathy. Insights provided by comparisons of kindreds with distinct and identical beta-myosin heavy chain gene mutations. *Circulation* 89 22–32. 10.1161/01.CIR.89.1.22 8281650

[B13] Farrell E. T. Grimes A. C. de Lange W. J. Armstrong A. E. Ralphe J. C. (2017). Increased postnatal cardiac hyperplasia precedes cardiomyocyte hypertrophy in a model of hypertrophic cardiomyopathy. *Front. Physiol.* 8:414. 10.3389/fphys.2017.00414 28659827 PMC5470088

[B14] Flegel C. Manteniotis S. Osthold S. Hatt H. Gisselmann G. (2013). Expression profile of ectopic olfactory receptors determined by deep sequencing. *PLoS One* 8:e55368. 10.1371/journal.pone.0055368 23405139 PMC3566163

[B15] Frank D. Frey N. (2011). Cardiac Z-disc signaling network. *J. Biol. Chem.* 286 9897–9904. 10.1074/jbc.R110.174268 21257757 PMC3060542

[B16] Granados-Riveron J. T. Brook J. D. (2012). Formation, contraction, and mechanotransduction of myofribrils in cardiac development: clues from genetics. *Biochem. Res. Int.* 2012:504906. 10.1155/2012/504906 22720160 PMC3376475

[B17] Harris S. P. Bartley C. R. Hacker T. A. McDonald K. S. Douglas P. S. Greaser M. L. (2002). Hypertrophic cardiomyopathy in cardiac myosin binding protein-C knockout mice. *Circ. Res.* 90 594–601. 10.1161/01.RES.0000012222.70819.64 11909824

[B18] Hoshijima M. (2006). Mechanical stress-strain sensors embedded in cardiac cytoskeleton: Z disk, titin, and associated structures. *Am. J. Physiol. Heart Circ. Physiol.* 290 H1313–H1325. 10.1152/ajpheart.00816.2005 16537787 PMC3241960

[B19] Huang H. T. Brand O. M. Mathew M. Ignatiou C. Ewen E. P. McCalmon S. A. (2006). Myomaxin is a novel transcriptional target of MEF2A that encodes a Xin-related alpha-actinin-interacting protein. *J. Biol. Chem.* 281 39370–39379. 10.1074/jbc.M603244200 17046827

[B20] Huang L. Wu K. H. Zhang L. Wang Q. Tang S. Wu Q. (2018). Critical Roles of Xirp proteins in cardiac conduction and their rare variants identified in sudden unexplained nocturnal death syndrome and Brugada syndrome in Chinese Han population. *J. Am. Heart Assoc.* 7:e006320. 10.1161/JAHA.117.006320 29306897 PMC5778954

[B21] Jiang J. Burgon P. G. Wakimoto H. Onoue K. Gorham J. M. O’Meara C. C. (2015). Cardiac myosin binding protein C regulates postnatal myocyte cytokinesis. *Proc. Natl. Acad. Sci. U.S.A.* 112 9046–9051. 10.1073/pnas.1511004112 26153423 PMC4517252

[B22] Jovancevic N. Dendorfer A. Matzkies M. Kovarova M. Heckmann J. C. Osterloh M. (2017). Medium-chain fatty acids modulate myocardial function via a cardiac odorant receptor. *Basic Res. Cardiol.* 112:13. 10.1007/s00395-017-0600-y 28116519 PMC5258789

[B23] Jung-Ching Lin J. Gustafson-Wagner E. A. Sinn H. W. Choi S. Jaacks S. M. Wang D. Z. (2005). Structure, expression, and function of a novel intercalated disc protein, Xin. *J. Med. Sci.* 25 215–222. 16708114 10.1901/jaba.2005.25-215 PMC1458968

[B24] Kaimal V. Bardes E. E. Tabar S. C. Jegga A. G. Aronow B. J. (2010). ToppCluster: a multiple gene list feature analyzer for comparative enrichment clustering and network-based dissection of biological systems. *Nucleic Acids Res.* 38 W96–W102. 10.1093/nar/gkq418 20484371 PMC2896202

[B25] Kamdar F. Klaassen Kamdar A. Koyano-Nakagawa N. Garry M. G. Garry D. J. (2015). Cardiomyopathy in a dish: using human inducible pluripotent stem cells to model inherited cardiomyopathies. *J. Cardiac. Fail.* 21 761–770. 10.1016/j.cardfail.2015.04.010 25934595 PMC4554831

[B26] Kebir S. Orfanos Z. Schuld J. Linhart M. Lamberz C. van der Ven P. F. (2016). Sarcomeric lesions and remodeling proximal to intercalated disks in overload-induced cardiac hypertrophy. *Exp. Cell Res.* 348 95–105. 10.1016/j.yexcr.2016.09.008 27639425

[B27] Kley R. A. Maerkens A. Leber Y. Theis V. Schreiner A. van der Ven P. F. (2013). A combined laser microdissection and mass spectrometry approach reveals new disease relevant proteins accumulating in aggregates of filaminopathy patients. *Mol. Cell. Proteomics* 12 215–227. 10.1074/mcp.M112.023176 23115302 PMC3536902

[B28] Lin B. Govindan S. Lee K. Zhao P. Han R. Runte K. E. (2013). Cardiac myosin binding protein-C plays no regulatory role in skeletal muscle structure and function. *PLoS One* 8:e69671. 10.1371/journal.pone.0069671 23936073 PMC3729691

[B29] Long P. A. Larsen B. T. Evans J. M. Olson T. M. (2015). Exome sequencing identifies pathogenic and modifier mutations in a child with sporadic dilated cardiomyopathy. *J. Am. Heart Assoc.* 4:e002443. 10.1161/JAHA.115.002443 26656454 PMC4845292

[B30] Lyon R. C. Zanella F. Omens J. H. Sheikh F. (2015). Mechanotransduction in cardiac hypertrophy and failure. *Circ. Res.* 116 1462–1476. 10.1161/CIRCRESAHA.116.304937 25858069 PMC4394185

[B31] Maron B. J. Towbin J. A. Thiene G. Antzelevitch C. Corrado D. Arnett D. (2006). Contemporary definitions and classification of the cardiomyopathies: an american heart association scientific statement from the council on clinical cardiology, heart failure and transplantation committee; quality of care and outcomes research and functional genomics and translational biology interdisciplinary working groups; and council on epidemiology and prevention. *Circulation* 113 1807–1816. 10.1161/CIRCULATIONAHA.106.174287 16567565

[B32] McCalmon S. A. Desjardins D. M. Ahmad S. Davidoff K. S. Snyder C. M. Sato K. (2010). Modulation of angiotensin II-mediated cardiac remodeling by the MEF2A target gene Xirp2. *Circ. Res.* 106 952–960. 10.1161/CIRCRESAHA.109.209007 20093629 PMC2858324

[B33] Merkulov S. Chen X. Chandler M. P. Stelzer J. E. (2012). In vivo cardiac myosin binding protein C gene transfer rescues myofilament contractile dysfunction in cardiac myosin binding protein C null mice. *Circ. Heart Fail.* 5 635–644. 10.1161/CIRCHEARTFAILURE.112.968941 22855556 PMC4860813

[B34] Moolman J. C. Corfield V. A. Posen B. Ngumbela K. Seidman C. Brink P. A. (1997). Sudden death due to troponin T mutations. *J. Am. Coll. Cardiol.* 29 549–555. 10.1016/S0735-1097(96)00530-X 9060892

[B35] Nixon B. R. Williams A. F. Glennon M. S. de Feria A. E. Sebag S. C. Baldwin H. S. (2017). Alterations in sarcomere function modify the hyperplastic to hypertrophic transition phase of mammalian cardiomyocyte development. *JCI Insight* 2:e90656. 10.1172/jci.insight.90656 28239655 PMC5313062

[B36] Olivotto I. Girolami F. Ackerman M. J. Nistri S. Bos J. M. Zachara E. (2008). Myofilament protein gene mutation screening and outcome of patients with hypertrophic cardiomyopathy. *Mayo Clin. Proc.* 83 630–638. 10.4065/83.6.630 18533079

[B37] Richard P. Charron P. Carrier L. Ledeuil C. Cheav T. Pichereau C. (2003). Hypertrophic cardiomyopathy: distribution of disease genes, spectrum of mutations, and implications for a molecular diagnosis strategy. *Circulation* 107 2227–2232. 10.1161/01.CIR.0000066323.15244.54 12707239

[B38] Semsarian C. Ingles J. Maron M. S. Maron B. J. (2015). New perspectives on the prevalence of hypertrophic cardiomyopathy. *J. Am. Coll. Cardiol.* 65 1249–1254. 10.1016/j.jacc.2015.01.019 25814232

[B39] Senbonmatsu T. Saito T. Landon E. J. Watanabe O. Price E. Jr. Roberts R. L. (2003). A novel angiotensin II type 2 receptor signaling pathway: possible role in cardiac hypertrophy. *EMBO J.* 22 6471–6482. 10.1093/emboj/cdg637 14657020 PMC291832

[B40] Swope D. Cheng L. Gao E. Li J. Radice G. L. (2012). Loss of cadherin-binding proteins beta-catenin and plakoglobin in the heart leads to gap junction remodeling and arrhythmogenesis. *Mol. Cell. Biol.* 32 1056–1067. 10.1128/MCB.06188-11 22252313 PMC3295003

[B41] Takeuchi S. Takagishi Y. Yasui K. Murata Y. Toyama J. Kodama I. (2000). Voltage-gated K( + )Channel, Kv4.2, localizes predominantly to the transverse-axial tubular system of the rat myocyte. *J. Mol. Cell. Cardiol.* 32 1361–1369. 10.1006/jmcc.2000.1172 10860776

[B42] Toib A. Zhang C. Borghetti G. Zhang X. Wallner M. Yang Y. (2017). Remodeling of repolarization and arrhythmia susceptibility in a myosin binding protein C knockout mouse model. *Am. J. Physiol.* 31:ajheart.00167. 10.1152/ajpheart.00167.2017 28646025 PMC5625176

[B43] Tzatzalos E. Abilez O. J. Shukla P. Wu J. C. (2016). Engineered heart tissues and induced pluripotent stem cells: macro- and microstructures for disease modeling, drug screening, and translational studies. *Adv. Drug Deliv. Rev.* 96 234–244. 10.1016/j.addr.2015.09.010 26428619 PMC4698222

[B44] Vermij S. H. Abriel H. van Veen T. A. (2017). Refining the molecular organization of the cardiac intercalated disc. *Cardiovasc. Res.* 113 259–275. 10.1093/cvr/cvw259 28069669

[B45] Wang N. Frank G. D. Ding R. Tan Z. Rachakonda A. Pandolfi P. P. (2012). Promyelocytic leukemia zinc finger protein activates GATA4 transcription and mediates cardiac hypertrophic signaling from angiotensin II receptor 2. *PLoS One* 7:e35632. 10.1371/journal.pone.0035632 22558183 PMC3338737

[B46] Wang Q. Lin J. J. (2013). Xin scaffolding proteins and arrhythmias. *J. Cardiol. Clin. Res.* 1:1011. 24734257 PMC3984570

[B47] Wang Q. Lin J. L. Erives A. J. Lin C. I. Lin J. J. (2014). New insights into the roles of Xin repeat-containing proteins in cardiac development, function, and disease. *Int. Rev. Cell Mol. Biol.* 310 89–128. 10.1016/B978-0-12-800180-6.00003-7 24725425 PMC4857591

[B48] Wang Q. Lin J. L. Reinking B. E. Feng H. Z. Chan F. C. Lin C. I. (2010). Essential roles of an intercalated disc protein, mXinbeta, in postnatal heart growth and survival. *Circ. Res.* 106 1468–1478. 10.1161/CIRCRESAHA.109.212787 20360251 PMC2872156

[B49] Wang Q. Lin J. L. Wu K. H. Wang D. Z. Reiter R. S. Sinn H. W. (2012). Xin proteins and intercalated disc maturation, signaling and diseases. *Front. Biosci.* 17 2566–2593. 22652799 10.2741/4072 PMC3368345

[B50] Zou Y. Akazawa H. Qin Y. Sano M. Takano H. Minamino T. (2004). Mechanical stress activates angiotensin II type 1 receptor without the involvement of angiotensin II. *Nat. Cell Biol.* 6 499–506. 10.1038/ncb1137 15146194

