# Supplementary material for: Transcriptome Analysis of Cardiac Hypertrophic Growth in MYBPC3-Null Mice Suggests Early Responders in Hypertrophic Remodeling
Source: Front Physiol. 2018 Oct 25;9:1442. doi: 10.3389/fphys.2018.01442 (PMC6210548; doi:10.3389/fphys.2018.01442)
Supplement: Supplementary file 1 [file Data_Sheet_1.pdf]

Supplemental Methods

RT-qPCR

Heart tissue was homogenized using TRIzol reagent (Ambion) and an appropriate amount of chloroform (MP Biomedicals) was added, according to the manufacturer's instructions. After mixing, incubation, and centrifugation, the aqueous phase containing RNA was collected and treated with RNase-free DNase I (Qiagen) for 30 minutes before being treated using the Mini Total RNA Kit (Blood/Cultured Cells; IBI Scientific). 500ng total RNA was reverse transcribed into first-strand cDNA with the iScript Reverse Transcription Supermix for RT-qPCR (Bio-Rad) following manufacturer's protocols. 10ng of resulting cDNA was used for qPCR analysis, using the TaqMan Gene Expression Master Mix (Applied Biosystems). Real-time monitoring of TaqMan fluorescence was performed on the Stratagene Mx3005P qPCR system (Agilent Technologies). An initial activation step of 95°C for 10 minutes was followed by 40 cycles of 15 seconds denaturation and 60 seconds annealing/extension. Data was analyzed in Excel, using the ΔΔCT method, with *Gapdh*/*Actb* used as housekeeping control genes. TaqMan assay IDs used were: *Gapdh* (Mm99999915_g1 and MmPT.39a.1(IDT)), *Actb* (Mm00607939_s1), *Xirp2* (Mm01335343_m1), *Myh6* (Mm00440359_m1), *Myh7* (Mm00600555_m1), *Tgfb2* (Mm00436955_m1), *Mybpc3* (Mm00435104_m1), *Actc1* (Mm01333821_m1), *Cmya5* (Mm01282680_m1), *Zbtb16* (Mm01176868_m1), *Itgb6* (Mm01269869), *Ankrd23* (Mm00463265_m1), and *Kcnd2* (Mm01161732_m1).

Cryopreservation of whole hearts

Hearts were harvested from WT and cMyBP-C$^{-/-}$ pups at PND1, rinsed in DPBS (Thermo Fisher Scientific), and incubated in filtered 30% sucrose (in DPBS) solution for at least one hour, until hearts equilibrated and sank. Hearts were then transferred to a 1:1 mixture of optimal cutting temperature (OCT) compound (Tissue-Tek) and 30% sucrose solution in DPBS for one hour. Hearts were positioned ventral side up in pre-cooled cryomolds (Tissue-Tek) resting on dry ice, covered with OCT compound, and frozen. Hearts in OCT were then stored at -80˚C until sectioned.
