# Supplementary material for: Transcriptome Analysis of Cardiac Hypertrophic Growth in MYBPC3-Null Mice Suggests Early Responders in Hypertrophic Remodeling
Source: Front Physiol. 2018 Oct 25;9:1442. doi: 10.3389/fphys.2018.01442 (PMC6210548; doi:10.3389/fphys.2018.01442)
Supplement: Supplementary file 2 [file Data_Sheet_2.pdf]

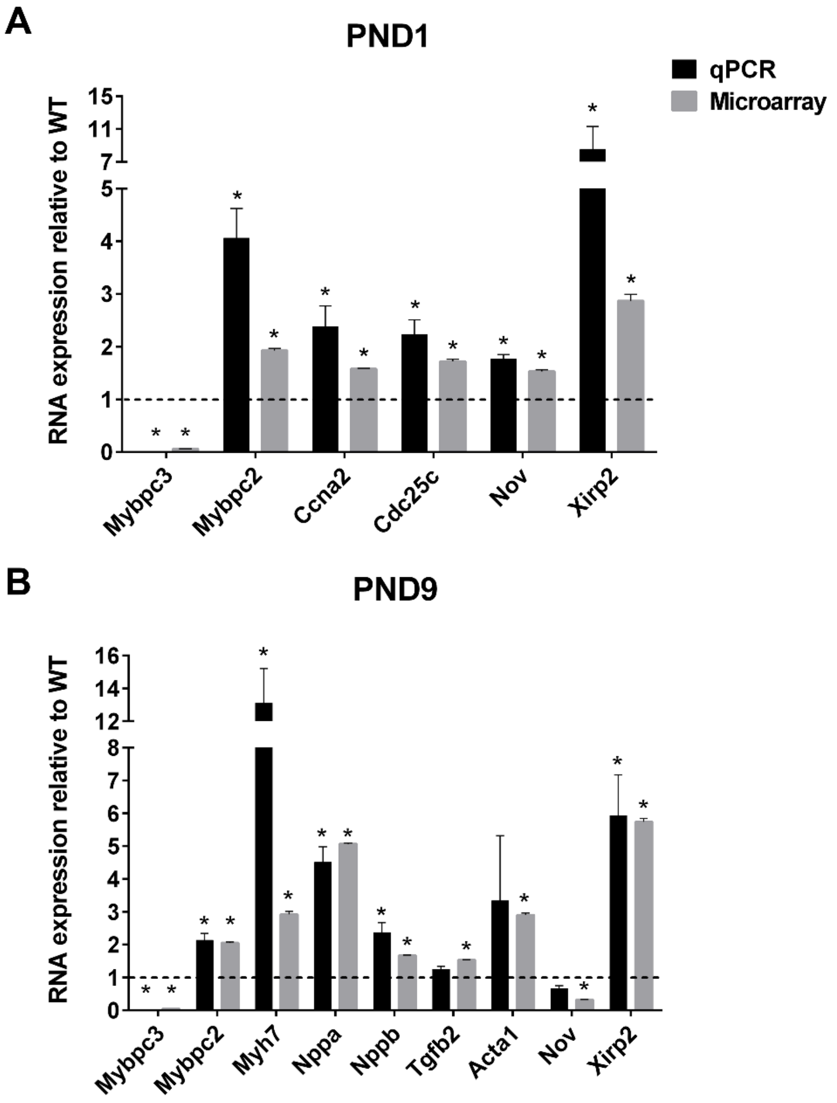

Supplemental Figure 1. Validation of Microarray using qPCR. RNA expression levels in cMyBP-C$^{-/-}$ left ventricles relative to WT (normalized to 1 for each gene, dashed line) at PND1 (A) and PND9 (B) determined by microarray and validated using qPCR. *Mybpc3*, myosin binding protein C, cardiac, *Mybpc2*, myosin binding protein C, fast type; *Ccna2*, cyclin A2; *Cdc25c*, cell division cycle 25c; *Nov*, nephroblastoma overexpressed; *Xirp2*, xin actin binding repeat containing 2; *Myh7*, myosin heavy chain 7; *Nppa*, natriuretic peptide A; *Nppb*, natriuretic peptide B; *Tgfb2*, transforming growth factor beta 2; *Acta1*, actin, alpha 1, skeletal muscle. * $\geq$ 1.5 fold change over WT and $p < 0.05$. n=5 (qPCR), n=7 (microarray).

Supplemental Table 1: Genes regulated ≥ 2 fold exclusively in physiologic growth (WTΔPND1-PND9)

Gene Name	Gene ID	Direction	Fold change	p-value
EF hand domain containing 1	Efhd1	Up	3.8	7.41E-05
Tripartite motif-containing 12	Trim12a	Up	3.4	1.41E-04
Chemokine (C-C motif) ligand 21A	Ccl21b	Up	3.0	2.76E-04
Peptidase inhibitor 16	Pi16	Up	2.6	6.51E-05
Microfibrillar associated protein 5	Mfap5	Up	2.6	1.54E-03
myosin light chain kinase family, member 4	Mylk4	Up	2.5	6.25E-03
Rho GTPase activating protein 20	Arhgap20	Up	2.5	4.23E-04
Carbonyl reductase 2	Cbr2	Up	2.4	4.68E-03
RIKEN cDNA 1700019D03 gene	1700019D03Rik	Up	2.4	6.02E-04
Sema domain, immunoglobulin domain (Ig), short basic domain, secreted, (semaphorin) 3C	Sema3c	Up	2.3	8.72E-05
Tetraspanin 13	Tspan13	Up	2.3	6.18E-05
Membrane metallo endopeptidase	Mme	Up	2.3	3.38E-04
Chemokine (C-X-C motif) receptor 4	Cxcr4	Up	2.2	5.33E-04
Adamts-like 3	Adamtsl3	Up	2.2	1.44E-04
Dual oxidase maturation factor 1	Duoxa1	Up	2.2	5.10E-05
Phospholipase A2, group V	Pla2g5	Up	2.2	1.28E-03
MAS-related GPR, member H	Mrgprh	Up	2.1	4.57E-03
Purinergic receptor P2Y, G-protein coupled, 14	P2ry14	Up	2.1	1.48E-04
GPI-anchored membrane protein CDw108 precursor	Sema7a	Up	2.1	3.26E-03
LIM and senescent cell antigen like domains 2	Lims2	Up	2.0	1.27E-03
Leucine-rich repeat LGI family, member 1	Lgi1	Up	2.0	1.78E-03
Tetraspanin 7	Tspan7	Up	2.0	3.59E-04
Myosin, heavy polypeptide 7, cardiac muscle, beta	Myh7	Down	4.7	4.70E-03
Natriuretic peptide precursor type A	Nppa	Down	3.6	6.01E-04
Dopa decarboxylase	Ddc	Down	2.6	6.57E-03
Procollagen-proline, 2-oxoglutarate 4-dioxygenase (proline 4-hydroxylase), alpha polypeptide III	P4ha3	Down	2.4	1.87E-04
Myosin, light polypeptide 9, regulatory	Myl9	Down	2.2	3.55E-04
Thrombospondin 4	Thbs4	Down	2.2	6.29E-04
Colorectal Neoplasia Differentially Expressed	Crnde	Down	2.2	5.33E-03
Insulin-like growth factor binding protein 3	Igfbp3	Down	2.0	6.19E-05

Supplemental Table 2: Genes regulated ≥ 2 fold exclusively in hypertrophic growth ($cMyBP\text{-}C^{-/-}$ ΔPND1-PND9)

Gene Name	Gene ID	Direction	Fold change	p-value
Uncoupling protein 3 (mitochondrial, proton carrier)	Ucp3	Up	5.5	2.72E-06
Aquaporin 8	Aqp8	Up	4.8	2.91E-06
Secreted frizzled-related protein 2	Sfrp2	Up	4.0	6.88E-05
Serine (or cysteine) peptidase inhibitor, clade A, member 3K	Serpina3n	Up	3.4	4.07E-03
Actin, alpha 1, skeletal muscle	Acta1	Up	3.3	4.54E-04
Phospholipase B Domain Containing 1	Plbd1	Up	3.1	5.72E-05
Prostate stem cell antigen	Psca	Up	2.9	2.15E-02
Keratin 18	Krt18	Up	2.8	5.31E-04
Integrin, beta-like 1	Itgbl1	Up	2.7	3.67E-05
Elastin	Eln	Up	2.6	4.55E-06
Pwcr1	Snord116@	Up	2.5	1.70E-05
Keratin 8	Krt8	Up	2.4	1.15E-04
Sushi, von Willebrand factor type A, EGF and pentraxin domain containing 1	Svep1	Up	2.4	3.07E-05
Glycoprotein m6b	Gpm6b	Up	2.4	8.63E-08
Mesenchyme homeobox 1	Meox1	Up	2.4	2.67E-05
small nucleolar RNA, C/D box 35B	Snord35b	Up	2.4	1.25E-02
Transmembrane protein 171	Tmem171	Up	2.4	1.13E-04
Frem2 mRNA for Fras1 related extracellular matrix protein 2	Frem2	Up	2.4	1.30E-07
Solute carrier family 25, member 34	Slc25a34	Up	2.3	2.57E-04
Matrin 3	Snora74a	Up	2.3	2.48E-02
MPS1 gene and mRNA, 3end	Mpeg1	Up	2.3	1.04E-05
Cartilage intermediate layer protein, nucleotide pyrophosphohydrolase	Cilp	Up	2.2	6.49E-05
Angiopoietin-like 4	Angptl4	Up	2.2	1.35E-02
Platelet-derived growth factor receptor-like	Pdgfrl	Up	2.2	2.26E-06
Fin bud initiation factor homolog (zebrafish)	Fibin	Up	2.2	3.17E-05
Small nucleolar RNA, C/D Box 14B	Snord15b	Up	2.1	2.80E-02
Peptidase inhibitor 15	Pi15	Up	2.1	3.62E-04
Olfactory receptor 1396	Olfr1396	Up	2.1	8.08E-05
Basic helix-loop-helix family, member e41	Bhlhe41	Up	2.1	3.88E-04
A disintegrin-like and metallopeptidase (reprolysin type) with thrombospondin type 1 motif, 20	Adamts20	Up	2.1	4.41E-06
Carboxypeptidase X 2 (M14 family)	Cpxm2	Up	2.0	8.54E-06
CD52 antigen	Cd52	Up	2.0	4.69E-03
Apolipoprotein B mRNA editing enzyme, catalytic polypeptide 1, transcript variant 1	Apobec1	Up	2.0	1.09E-04
Proline rich basic protein 1	Prob1	Up	2.0	6.59E-06
Potassium channel, subfamily T, member 2	Kcnt2	Up	2.0	9.03E-05
Complement component factor h	Cfh	Up	2.0	1.17E-03

Matrix Gla protein	Mgp	Up	2.0	1.44E-03
Tubulin, alpha 4A	Tuba4a	Up	2.0	7.27E-03
Otoancorin	Otoa	Up	2.0	8.52E-08
Laminin, alpha 2	Lama2	Up	2.0	1.38E-06
Carboxypeptidase A3, mast cell	Cpa3	Down	5.0	1.43E-06
Lymphatic vessel endothelial hyaluronan receptor 1	Lyve1	Down	3.7	5.53E-06
Relaxin/insulin-like family peptide receptor 1	Rxfp1	Down	2.8	7.92E-03
Zinc Finger and BTB domain containing 16	Zbtb16	Down	2.7	4.33E-03
Interferon-induced protein 44	Ifi44	Down	2.4	3.85E-03
Growth associated protein 43	Gap43	Down	2.4	2.73E-05
Tryptase beta 2	Tpsb2	Down	2.3	2.44E-05
Aquaporin 4	Aqp4	Down	2.3	3.08E-04
Mitochondria localized glutamic acid rich protein	Mgarp	Down	2.2	8.37E-04
Histidine decarboxylase	Hdc	Down	2.2	2.76E-03
Carboxypeptidase A1	Cpa1	Down	2.2	3.60E-03
Dipeptidylpeptidase 4	Dpp4	Down	2.1	2.21E-05
Uroplakin Ib	Upk1b	Down	2.1	1.45E-04
Paternally expressed 10, transcript variant 1	Peg10	Down	2.1	4.32E-05
Cilia and flagella associated protein 69	Cfap69	Down	2.1	1.13E-02
Uroplakin 3B	Upk3b	Down	2.0	5.88E-04
Small nucleolar RNA, C/D Box 115 cluster	Snord115	Down	2.0	8.21E-03
Glutathione S-transferase, alpha 4	Gsta4	Down	2.0	3.42E-05
Cyclin-dependent kinase inhibitor 1C (P57)	Cdkn1c	Down	2.0	3.25E-06
Proline and serine rich 2	Proser2	Down	2.0	3.50E-04
Solute carrier family 39 (metal ion transporter), member 8	Slc39a8	Down	2.0	3.69E-04

Supplemental Table 3: Genes regulated ≥ 2 fold in both physiologic (WT ΔPND1-PND9) and hypertrophic growth (cMyBP-C$^{-/-}$ΔPND1-PND9)

Gene Name	Gene ID	Direction	WT Ratio	p-value	-/- Ratio	p-value	Fold change ratio (-/-)/WT
Xin Actin Binding Repeat Containing 2	Xirp2	Up	3.1	2.55E-03	6.1	7.64E-05	2.0
Tubulin, alpha 8	Tuba8	Up	2.8	3.03E-02	5.1	1.35E-04	1.8
Histocompatibility 2, class II antigen A, alpha	H2-Aa	Up	2.1	6.35E-05	3.9	7.72E-07	1.8
Glycosyltransferase 8 domain containing 4	Gxylt2	Up	2.0	8.94E-04	3.5	2.89E-10	1.8
ATP-binding cassette, sub-family A (ABC1), member 8a	Abca8a	Up	2.2	2.73E-03	3.6	3.01E-07	1.6
Aquaporin 7	Aqp7	Up	2.6	1.56E-03	4.2	3.08E-05	1.6
Procollagen C-endopeptidase enhancer 2	Pcolce2	Up	4.4	1.38E-03	6.8	9.17E-07	1.6
CD74 antigen	Cd74	Up	2.0	7.74E-04	3.0	1.41E-06	1.5
STEAP family member 4	Steap4	Up	3.1	1.07E-03	4.5	2.81E-08	1.5
Proline arginine-rich end leucine-rich repeat	Prelp	Up	2.2	2.56E-03	3.2	5.43E-09	1.5
C-type lectin domain family 12, member a	Clec12a	Up	2.0	1.11E-03	2.8	4.56E-06	1.4
Kelch domain containing 8A	Klhdc8a	Up	2.2	4.83E-04	3.1	1.06E-06	1.4
Purine-selective Na+ nucleoside cotransporter	Slc28a2	Up	2.2	1.21E-04	2.9	1.03E-07	1.4
Ectodysplasin A2 isoform receptor	Eda2r	Up	2.0	1.20E-03	2.6	9.12E-06	1.3
Microfibrillar-associated protein 4	Mfap4	Up	2.3	1.33E-03	3.0	5.16E-09	1.3
Apelin receptor	Aplnr	Up	3.0	7.83E-05	3.8	7.89E-08	1.3
Extracellular matrix protein 2, female organ and adipocyte specific	Ecm2	Up	2.2	1.87E-04	2.7	1.30E-04	1.3
RIKEN cDNA 2310040G07 gene	2310040G07Rik	Up	2.2	2.29E-03	2.8	3.12E-07	1.3
Creatine kinase, mitochondrial 2	Ckmt2	Up	2.3	1.58E-04	2.9	3.44E-07	1.3
IFGP2	Fcrls	Up	2.2	7.27E-04	2.8	4.40E-05	1.3
Ras association (RalGDS/AF-6) domain family (N-terminal) member 9	Rassf9	Up	2.0	2.71E-04	2.5	6.31E-06	1.2
Chemokine (C-X3-C) receptor 1	Cx3cr1	Up	2.0	2.03E-03	2.4	6.74E-05	1.2
Ectonucleotide pyrophosphatase/ phosphodiesterase 3	Enpp3	Up	2.2	1.39E-04	2.6	5.98E-06	1.2
Interferon inducible GTPase 1	Iigp1	Up	2.0	2.84E-03	2.5	1.40E-04	1.2
Mesenchyme homeobox 2	Meox2	Up	2.0	5.88E-03	2.4	2.67E-05	1.2
Refilin B	Rflnb	Up	2.6	5.74E-04	3.2	4.24E-08	1.2
Cytoglobin	Cygb	Up	2.0	8.02E-04	2.3	1.41E-07	1.2
Androgen receptor	Ar	Up	2.1	1.07E-03	2.5	3.34E-06	1.2
Lumican	Lum	Up	2.6	1.97E-05	2.9	4.33E-06	1.1
Emr1	Adgre1	Up	2.0	2.39E-03	2.3	3.74E-05	1.1
RIKEN cDNA 4930578C19 gene	4930578C19Rik	Up	2.3	1.16E-03	2.6	2.64E-07	1.1
CDNA clone	BC022713	Up	2.8	1.82E-03	3.1	4.87E-06	1.1
Plexin domain containing 2	Plxdc2	Up	2.3	2.50E-04	2.5	1.40E-07	1.1
Regulator of G-protein signaling 5	Rgs5	Up	3.2	1.29E-03	3.6	1.95E-07	1.1
Hypermethylated in cancer 1	Hic1	Up	2.1	8.31E-04	2.3	6.24E-07	1.1
Regulator of G-protein signaling 4	Rgs4	Up	2.2	2.03E-03	2.4	4.09E-06	1.1
Tumor necrosis factor (ligand) superfamily, member 10	Tnfsf10	Up	2.9	6.81E-05	3.1	5.56E-06	1.1

Secretoglobin, family 1C, member 1	Scgb1c1	Up	4.2	1.82E-03	4.5	4.07E-06	1.1
Immunoglobulin superfamily containing leucine-rich repeat	Islr	Up	2.8	1.23E-04	3.0	1.47E-06	1.1
Collagen, type XV, alpha 1	Col15a1	Up	2.4	8.64E-04	2.6	1.16E-06	1.1
Ankyrin repeat domain 23	Ankrd23	Up	2.2	2.94E-03	2.3	4.52E-05	1.1
RIKEN cDNA 9930111J21 gene	Gm5431	Up	2.2	1.89E-04	2.3	8.60E-06	1.0
N-terminal EF-hand calcium binding protein 1	Necab1	Up	2.3	2.57E-04	2.4	4.80E-08	1.0
Paired related homeobox 1, transcript variant 1	Prrx1	Up	2.1	2.44E-04	2.2	2.68E-06	1.0
Malic enzyme 3, NADP(+)-dependent, mitochondrial	Me3	Up	2.4	1.37E-03	2.5	1.86E-06	1.0
Olfactory receptor 558	Olfr558	Up	2.0	1.41E-03	2.1	1.03E-03	1.0
small nucleolar RNA, C/D box 32A	Snord32a	Up	2.1	2.91E-04	2.2	1.84E-05	1.0
Lymphocyte antigen 6 complex, locus A	Ly6a	Up	2.3	2.00E-03	2.3	5.80E-03	1.0
Hemochromatosis type 2 (juvenile) (human homolog)	Hfe2	Up	2.3	4.81E-03	2.3	6.81E-04	1.0
Strain ILS microtubule binding protein tau	Mapt	Up	2.2	3.80E-05	2.2	6.21E-07	1.0
Fructose bisphosphatase 2	Fbp2	Up	2.4	8.87E-03	2.4	1.90E-04	1.0
Potassium inwardly-rectifying channel, subfamily J, member 8	Kcnj8	Up	2.2	5.50E-04	2.1	1.33E-06	1.0
Protocadherin 17	Pcdh17	Up	2.1	3.00E-04	2.1	8.85E-07	1.0
DNA segment, Chr 4, Brigham & Womens Genetics 0951 expressed	Lurap1l	Up	2.0	9.69E-05	2.0	1.35E-05	1.0
Fibroblast activation protein	Fap	Up	3.4	3.95E-04	3.3	3.36E-06	1.0
small nucleolar RNA, C/D box 33	Snord33	Up	2.5	1.11E-02	2.4	6.82E-03	1.0
Small secreted protein interferon-induced	AW112010	Up	4.6	1.72E-04	4.5	8.96E-06	1.0
Strain ICR inducible nitric oxide synthase	Nos2	Up	2.1	6.26E-04	2.1	1.13E-07	1.0
Interferon activated gene 203	Ifi203	Up	2.3	4.45E-03	2.2	9.50E-03	1.0
Integrin alpha 7	Itga7	Up	2.2	2.86E-03	2.1	1.99E-05	1.0
Integrin beta 6	Itgb6	Up	3.4	3.48E-03	3.2	5.80E-06	0.9
Lymphocyte differentiation antigen Ly-6C.2	Ly6c1	Up	2.3	4.41E-04	2.2	2.90E-03	0.9
Leucine rich repeat containing 55	Lrrc55	Up	2.6	5.55E-05	2.4	4.25E-08	0.9
Tripartite motif-containing 72	Trim72	Up	2.1	5.04E-03	2.0	5.63E-04	0.9
Lymphocyte antigen 6 complex, locus C1	Ly6c1	Up	2.5	3.80E-04	2.3	1.88E-03	0.9
Ankyrin repeat domain-containing SOCS box protein Asb-14	Asb14	Up	2.9	7.01E-04	2.7	8.64E-05	0.9
Junction adhesion molecule 2	Jam2	Up	2.7	3.91E-04	2.2	8.51E-05	0.8
Family with sequence similarity 70, member B	Tmem255b	Up	2.5	5.86E-05	2.0	1.09E-05	0.8
SPARC related modular calcium binding 2	Smoc2	Up	2.4	3.25E-04	2.0	1.60E-04	0.8
Prion protein dublet	Prnd	Up	2.5	5.95E-03	2.0	2.39E-04	0.8
Aldo-keto reductase family 1, member C14	Akr1c14	Up	2.9	1.19E-04	2.4	6.02E-05	0.8
Neural proliferation, differentiation and control gene 1	Entpd2	Up	3.0	1.44E-04	2.4	4.80E-06	0.8
Hyperpolarization-activated, cyclic nucleotide-gated K+ 2	Hcn2	Up	2.9	5.48E-04	2.3	4.43E-06	0.8
Phospholipid transfer protein	Pltp	Up	2.7	9.07E-05	2.1	3.32E-05	0.8
Leucine Rich Repeats And Transmembrane Domains 1	Lrtm1	Up	4.2	2.11E-04	3.3	1.29E-04	0.8

Ankyrin repeat domain-containing SOCS box protein Asb-15	Asb15	Up	2.7	4.52E-03	2.0	1.91E-03	0.8
Proprotein convertase subtilisin/kexin type 6	Pcsk6	Up	3.4	1.28E-04	2.3	1.35E-04	0.7
Vitronectin	Vtn	Up	5.0	2.57E-04	3.4	8.71E-06	0.7
C1q and tumor necrosis factor related protein 9	C1qtnf9	Up	3.7	8.87E-05	2.3	2.58E-03	0.6
Retinoid X receptor gamma	Rxrg	Up	5.1	2.66E-04	3.0	1.18E-05	0.6
Bone morphogenetic protein 10 precursor	Bmp10	Down	2.1	2.81E-02	4.1	6.03E-04	2.0
Delta-like 1 homolog (Drosophila)	Dlk1	Down	3.2	5.37E-04	5.5	2.24E-08	1.7
Inhibitory PAS domain protein	Hif3a	Down	2.5	4.59E-03	4.0	5.13E-05	1.6
Zinc finger protein	Zim1	Down	2.9	7.90E-04	4.5	5.83E-09	1.5
alcohol dehydrogenase 6B (class V)	Adh6b	Down	3.2	3.95E-03	4.8	2.21E-06	1.5
G protein-coupled receptor 126	Adgrg6	Down	2.5	2.00E-03	3.6	4.40E-07	1.4
Tuftelin 1	Tuft1	Down	2.2	3.06E-03	3.2	5.31E-04	1.4
Phospholipid Phosphatase Related 1	Plppr1	Down	2.0	9.14E-03	2.7	3.22E-04	1.4
Diacylglycerol kinase kappa	Dgkk	Down	2.1	1.44E-03	2.8	4.14E-05	1.3
Zinc Finger DBF-Type Containing 2	Zdbf2	Down	2.2	6.70E-04	2.8	6.17E-09	1.3
Solute carrier family 2 (facilitated glucose transporter), member 1	Slc2a1	Down	2.8	7.76E-04	3.6	1.76E-06	1.3
Aldolase C, fructose-bisphosphate	Aldoc	Down	3.3	5.68E-04	4.1	1.65E-06	1.3
Alpha-2-macroglobulin	A2m	Down	2.7	1.18E-03	3.4	2.65E-06	1.2
H19 fetal liver	H19	Down	2.5	5.94E-04	3.0	2.69E-07	1.2
Myosin, light polypeptide 7, regulatory	Myl7	Down	10.1	1.36E-03	12.0	3.82E-07	1.2
Olfactory receptor 608	Olfr608	Down	2.8	1.15E-03	3.3	1.62E-04	1.2
P-cadherin	Cdh3	Down	2.3	2.55E-03	2.6	6.94E-06	1.2
Insulin-like growth factor 2 mRNA binding protein 1	Igf2bp1	Down	2.2	1.28E-05	2.5	1.74E-08	1.2
MKIAA1690 protein	Tmem108	Down	2.1	4.78E-03	2.5	1.26E-06	1.2
Cbp/p300-interacting transactivator with Glu/Asp-rich carboxy-terminal domain 1	Cited1	Down	2.5	6.78E-04	2.8	7.10E-05	1.2
HES-related repressor protein 1 HERP1	Hey2	Down	4.2	3.04E-04	4.8	2.41E-07	1.1
Cytochrome P450, family 51	Cyp51	Down	2.0	8.84E-05	2.2	5.42E-06	1.1
Natriuretic peptide receptor 3	Npr3	Down	2.1	4.16E-03	2.3	1.75E-05	1.1
24-dehydrocholesterol reductase	Dhcr24	Down	2.6	1.12E-05	2.9	9.74E-07	1.1
Lost on transformation protein 1	Plagl1	Down	2.4	2.09E-03	2.6	3.96E-05	1.1
Troponin I, skeletal, slow 1	Tnni1	Down	2.0	3.97E-03	2.2	5.19E-03	1.1
Intermediate filament tail domain containing 1	Lmntd1	Down	3.4	6.54E-05	3.6	2.08E-07	1.1
EF-9 polyadenylation variant II	Oit3	Down	3.5	1.14E-04	3.7	1.31E-07	1.1
Flavin containing monooxygenase 2	Fmo2	Down	2.0	8.90E-03	2.1	3.69E-04	1.0
Isopentenyl-diphosphate delta isomerase	Idi1	Down	3.0	1.42E-04	3.1	1.35E-07	1.0
Fras1 protein	Fras1	Down	2.1	2.53E-04	2.2	9.88E-06	1.0
Brain expressed X-linked protein 1	Bex1	Down	3.6	6.06E-04	3.7	1.13E-06	1.0
Keratin 7	Krt7	Down	2.0	9.38E-03	2.1	9.86E-05	1.0
Cebelin	Fam163a	Down	2.2	1.70E-03	2.1	5.20E-05	1.0
Sterol-C4-methyl oxidase-like	Msmo1	Down	2.8	1.10E-04	2.7	1.09E-09	1.0
Bone morphogenetic protein 5	Bmp5	Down	2.2	5.58E-05	2.0	3.37E-04	0.9
Neural cell adhesion molecule 1, transcript variant 2	Ncam1	Down	2.5	5.17E-04	2.3	1.68E-04	0.9

Brain expressed gene 4	Bex4	Down	3.5	7.95E-04	3.1	1.90E-05	0.9
Insulin-like growth factor 2 mRNA binding protein 3	Igf2bp3	Down	2.3	1.30E-04	2.0	2.20E-06	0.9
Calsequestrin 1	Casq1	Down	2.5	1.83E-03	2.2	1.89E-05	0.9
Squalene epoxidase	Sqle	Down	2.7	5.73E-05	2.3	1.62E-07	0.9
SWI/SNF related, matrix associated, actin dependent regulator of chromatin, subfamily a, member 1	Smarca1	Down	2.6	8.83E-04	2.1	9.41E-06	0.8
Metallothionein 2	Mt2	Down	2.7	1.62E-02	2.2	9.79E-03	0.8
Putative serine protease 35	Prss35	Down	2.4	1.05E-02	2.0	2.95E-03	0.8
Calpain 6	Capn6	Down	3.4	9.86E-04	2.7	3.79E-06	0.8
UDP-GlcNAc:betaGal beta-1,3-N-acetylglucosaminyltransferase 5	B3gnt5	Down	2.8	1.04E-04	2.1	7.85E-05	0.8
Bone morphogenetic protein 7	Bmp7	Down	3.6	1.31E-05	2.7	9.56E-05	0.7
Myosin, light polypeptide 4	Myl4	Down	5.1	1.67E-03	3.7	3.89E-06	0.7
Alpha-2-HS-glycoprotein	Ahsg	Down	2.9	7.02E-03	2.0	1.05E-02	0.7
Calponin 1	Cnn1	Down	3.9	6.88E-05	2.4	2.18E-04	0.6

Supplemental Table 4: Genes Upregulated or Downregulated ≥ 2 Fold at PND9 in cMyBP-C$^{-/-}$ vs WT

Gene Name	Gene ID	Ratio	Direction	p-value	Gene Identifier
Osteopontin	Spp1	7.2	Up	1.60E-06	NM_009263
Keratin 18	Krt18	6.3	Up	2.84E-06	NM_010664
Prostate stem cell antigen	Psca	6.0	Up	4.78E-04	NM_028216
Xin Actin Binding Repeat Containing 2	Xirp2	5.7	Up	5.27E-05	NM_001024618
Aquaporin 8	Aqp8	5.3	Up	3.99E-07	NM_007474
Natriuretic peptide precursor type A	Nppa	5.1	Up	2.09E-05	NM_008725
Keratin 8	Krt8	3.9	Up	9.64E-06	NM_031170
Serine (or cysteine) peptidase inhibitor, clade A, member 3K	Serpina3k	3.8	Up	1.17E-03	NM_009252
Sulfated glycoprotein-2 isoform 2	Clu	3.6	Up	9.91E-08	NM_013492
Collagen, type VIII, alpha 1	Col8a1	3.5	Up	4.27E-06	NM_007739
Secreted frizzled-related protein 2	Sfrp2	3.4	Up	9.57E-05	NM_009144
Thrombospondin 4	Thbs4	2.9	Up	2.74E-04	NM_011582
Myosin, heavy polypeptide 7, cardiac muscle, beta	Myh7	2.9	Up	4.42E-02	NM_080728
Actin, alpha 1, skeletal muscle	Acta1	2.9	Up	6.46E-04	NM_009606
Small proline-rich protein 1A	Sprr1a	2.8	Up	1.04E-04	NM_009264
Calponin 1	Cnn1	2.7	Up	1.37E-03	NM_009922
Leprecan-like 1	Leprel1	2.7	Up	2.41E-05	NM_173379
Tropomyosin 2, beta	Tpm2	2.7	Up	1.12E-04	NM_009416
Glutamine synthetase	Glul	2.6	Up	2.31E-09	NM_008131
Angiopoietin-like 7	Angptl7	2.5	Up	1.23E-07	NM_001039554
Integrin, beta-like 1	Itgbl1	2.4	Up	1.38E-06	NM_145467
Tenascin C	Tnc	2.4	Up	1.87E-06	NM_011607
Glycoprotein (transmembrane) nmb	Gpnmb	2.3	Up	3.06E-05	NM_053110
Inhibin beta-A	Inhba	2.3	Up	1.58E-05	NM_008380
ADAMTS-like 2	Adamtsl2	2.3	Up	2.15E-05	NM_029981
Glutathione peroxidase 3, transcript variant 2	Gpx3	2.2	Up	2.28E-04	NM_001083929
A disintegrin-like and metallopeptidase (reprolysin type) with thrombospondin type 1 motif, 20	Adamts20	2.2	Up	1.31E-06	NM_177431
Insulin-like growth factor binding protein 3	Igfbp3	2.2	Up	2.15E-04	NM_008343
Cytochrome P450, family 1, subfamily b, polypeptide 1	Cyp1b1	2.2	Up	6.43E-04	NM_009994
Elastin	Eln	2.1	Up	3.50E-06	NM_007925
Dopachrome tautomerase	Dct	2.1	Up	6.89E-04	NM_010024
Angiotensin I converting enzyme (peptidyl-dipeptidase A)	Ace	2.1	Up	6.19E-04	NM_207624
Calsequestrin 1, nuclear gene encoding mitochondrial protein	Casq1	2.1	Up	2.54E-03	NM_009813
Myosin binding protein C, fast-type	Mybpc2	2.1	Up	5.51E-04	NM_178067
Integrin beta 1 binding protein 3	Itgb1bp3	2.1	Up	3.47E-03	NM_027120
Gamma-aminobutyric acid (GABA-A) receptor, subunit beta 3, transcript variant 1	Gabrb3	2.0	Up	4.53E-05	NM_008071

Serine (or cysteine) peptidase inhibitor, clade E, member 1	Serpine1	2.0	Up	5.83E-06	NM_008871
Guanine nucleotide binding protein, alpha O (Gnao1), transcript variant A	Gnao1	2.0	Up	7.43E-07	NM_010308
SLIT and NTRK-like family, member 4	Slitrk4	2.0	Up	1.89E-05	NM_178740
Signal peptide, CUB domain, EGF-like 2	Scube2	2.0	Up	2.55E-07	NM_020052
Monoamine oxidase A	Maoa	2.0	Up	5.01E-06	NM_173740
Potassium voltage-gated channel, Isk-related subfamily, gene 3	Kcne3	2.0	Up	7.47E-03	NM_020574
Multimerin 1	Mmrn1	2.0	Down	2.81E-02	BC137623
Carboxypeptidase A3, mast cell	Cpa3	2.0	Down	4.03E-02	NM_007753
Solute carrier family 36 (proton/amino acid symporter), member 2	Slc36a2	2.0	Down	6.69E-06	NM_153170
Small membrane AKAP/Chromosome 2 open reading frame 88	C2orf88	2.0	Down	2.58E-03	NM_144953
Membrane metallo endopeptidase	Mme	2.0	Down	4.90E-03	NM_008604
Carbonyl reductase 2	Cbr2	2.0	Down	1.26E-02	NM_007621
Glutamine fructose-6-phosphate transaminase 2	Gfpt2	2.0	Down	7.81E-06	NM_013529
MAS-related GPR, member H	Mrgprh	2.0	Down	4.92E-03	NM_030726
Rho GTPase activating protein 36	Arhgap36	2.0	Down	7.39E-06	NM_001081123
Solute carrier family 1 (neuronal/epithelial high affinity glutamate transporter, system Xag), member 1	Slc1a1	2.0	Down	1.77E-04	NM_009199
Potassium inwardly-rectifying channel, subfamily J, member 3	Kcnj3	2.1	Down	1.62E-05	NM_008426
Dual oxidase maturation factor 1	Duoxa1	2.1	Down	1.02E-04	NM_145395
Rho GTPase activating protein 20	Arhgap20	2.1	Down	4.75E-03	NM_175535
Hexaribonucleotide binding protein 1	A2bp1	2.1	Down	2.75E-05	NM_021477
Ankyrin repeat domain-containing SOCS box protein	Asb15	2.2	Down	1.43E-02	NM_080847
Dehydrogenase/reductase (SDR family) member 7C	Dhrs7c	2.2	Down	1.36E-06	NM_001013013
Microfibrillar associated protein 5	Mfap5	2.4	Down	1.96E-03	NM_015776
Olfactory receptor 608	Olfr608	2.5	Down	2.89E-03	NM_146756
Aquaporin 4	Aqp4	2.7	Down	1.91E-05	NM_009700
Retinoid X receptor gamma	Rxrg	2.8	Down	8.40E-03	NM_009107
Phospholipase A2, group V	Pla2g5	2.8	Down	7.52E-05	NM_011110
Potassium voltage-gated channel, Shal-related family, member 2	Kcnd2	2.9	Down	1.55E-05	NM_019697
Nephroblastoma overexpressed gene	Nov	3.1	Down	1.41E-06	NM_010930
myosin light chain kinase family	-	3.2	Down	5.75E-04	10408543
EF hand domain containing 1	Efhd1	3.3	Down	3.58E-04	NM_028889
Myosin binding protein C, cardiac	Mybpc3	20.2	Down	7.52E-14	NM_008653

Supplemental Table 5: Genes Dysregulated in in cMyBP-C$^{-/-}$ Hearts at Mechanosensing Regions (Highlighted in Figure 4)

Gene Name	Gene ID	Ratio	Direction	PND	Location
Xin actin binding repeat containing 2	Xirp2	2.87	Up	1	ID[1, 2], costamere[3], z-disc[3, 4]
		5.74	Up	9	
Zinc finger and BTB domain containing 16	Zbtb16	2.40	Up	1	nucleus[5, 6]
Ankyrin Repeat Domain 23/ DARP/ MARP3	Ankrd23	1.70	Up	1	ID[7], sarcomere[7], nucleus[7]
PDZ and LIM domain 3/ (PDZ-1LIM)	Pdlim3	1.55	Up	9	ID[8], z-disc[8, 9]
Cardiomyopathy Associated 5/ Myospryn	Cmya5	1.57	Down	1	ID[10], costamere[10, 11], sarcomere[12]
Integrin beta 6	Itgb6	1.67	Down	1	costamere[13, 14]

ID, intercalated disc.

1. Wang Q, Lin JL, Reinking BE, Feng HZ, Chan FC, Lin CI, Jin JP, Gustafson-Wagner EA, Scholz TD, Yang B and Lin JJ. Essential roles of an intercalated disc protein, mXinbeta, in postnatal heart growth and survival. *Circulation research*. 2010;106:1468-78.
2. Wang Q, Lin JL, Wu KH, Wang DZ, Reiter RS, Sinn HW, Lin CI and Lin CJ. Xin proteins and intercalated disc maturation, signaling and diseases. *Frontiers in bioscience (Landmark edition)*. 2012;17:2566-93.
3. Huang HT, Brand OM, Mathew M, Ignatiou C, Ewen EP, McCalmon SA and Naya FJ. Myomaxin is a novel transcriptional target of MEF2A that encodes a Xin-related alpha-actinin-interacting protein. *The Journal of biological chemistry*. 2006;281:39370-9.
4. Eulitz S, Sauer F, Pelissier MC, Boisguerin P, Molt S, Schuld J, Orfanos Z, Kley RA, Volkmer R, Wilmanns M, Kirfel G, van der Ven PF and Furst DO. Identification of Xin-repeat proteins as novel ligands of the SH3 domains of nebulin and nebulette and analysis of their interaction during myofibril formation and remodeling. *Molecular biology of the cell*. 2013;24:3215-26.
5. Senbonmatsu T, Saito T, Landon EJ, Watanabe O, Price E, Jr., Roberts RL, Imboden H, Fitzgerald TG, Gaffney FA and Inagami T. A novel angiotensin II type 2 receptor signaling pathway: possible role in cardiac hypertrophy. *Embo j*. 2003;22:6471-82.
6. McLoughlin P, Ehler E, Carlile G, Licht JD and Schafer BW. The LIM-only protein DRAL/FHL2 interacts with and is a corepressor for the promyelocytic leukemia zinc finger protein. *The Journal of biological chemistry*. 2002;277:37045-53.
7. Miller MK, Bang ML, Witt CC, Labeit D, Trombitas C, Watanabe K, Granzier H, McElhinny AS, Gregorio CC and Labeit S. The muscle ankyrin repeat proteins: CARP, ankrd2/Arpp and DARP as a family of titin filament-based stress response molecules. *J Mol Biol*. 2003;333:951-64.
8. Pashmforoush M, Pomies P, Peterson KL, Kubalak S, Ross J, Jr., Hefti A, Aebi U, Beckerle MC and Chien KR. Adult mice deficient in actinin-associated LIM-domain protein reveal a developmental pathway for right ventricular cardiomyopathy. *Nature medicine*. 2001;7:591-7.
9. Henderson JR, Pomies P, Auffray C and Beckerle MC. ALP and MLP distribution during myofibrillogenesis in cultured cardiomyocytes. *Cell motility and the cytoskeleton*. 2003;54:254-65.
10. Kouloumenta A, Mavroidis M and Capetanaki Y. Proper Perinuclear Localization of the TRIM-like Protein Myospryn Requires Its Binding Partner Desmin. *Journal of Biological Chemistry*. 2007;282:35211-35221.
11. Durham JT, Brand OM, Arnold M, Reynolds JG, Muthukumar L, Weiler H, Richardson JA and Naya FJ. Myospryn is a direct transcriptional target for MEF2A that encodes a striated muscle, alpha-actinin-interacting, costamere-localized protein. *The Journal of biological chemistry*. 2006;281:6841-9.
12. Sarparanta J, Blandin G, Charton K, Vihola A, Marchand S, Milic A, Hackman P, Ehler E, Richard I and Udd B. Interactions with M-band titin and calpain 3 link myospryn (CMYA5) to tibial and limb-girdle muscular dystrophies. *The Journal of biological chemistry*. 2010;285:30304-15.
13. Chen Y-M, Li H, Fan Y, Zhang Q-J, Li X, Wu L-J, Chen Z-j, Zhu C and Qian L-M. Identification of differentially expressed lncRNAs involved in transient regeneration of the neonatal C57BL/6J mouse heart by next-generation high-throughput RNA sequencing. *Oncotarget*. 2017;8:28052-28062.
14. Hoshijima M. Mechanical stress-strain sensors embedded in cardiac cytoskeleton: Z disk, titin, and associated structures. *Am J Physiol Heart Circ Physiol*. 2006;290:H1313-25.
